# Acceptance or rejection of welfare migration—an experimental investigation

**DOI:** 10.1007/s43546-022-00356-6

**Published:** 2022-10-24

**Authors:** Jürgen Huber, Laura Hueber, Daniel Kleinlercher, Thomas Stöckl

**Affiliations:** 1 Department of Banking and Finance, University of Innsbruck, Universitätsstrasse 15, 6020 Innsbruck, Austria; 2 Department Business & Management, MCI The Entrepreneurial School, Universitätsstrasse 15, 6020 Innsbruck, Austria

**Keywords:** Migration, Public good game, Uniform contribution, Behavioral economics, C71, C92, D72, F22, H41, O15

## Abstract

We study decisions on welcoming or opposing welfare migration in a laboratory setting with two societies in which one subject can migrate from the poorer to the richer society, provided a majority in the richer society votes to allow that. In each society, subjects indicate their preference for a percentage contribution to a public pool. The median of these rates sets the contributions paid by everybody; a feature that results in high contribution rates with an average of 90%. Varying the multiplier with which contributions are magnified before redistribution to society members, and thus the expected gain/loss associated with migration, we find that subjects overwhelmingly welcome migrants if they expect an economic benefit, while most participants oppose migration if they would be negatively affected by it. Regarding participants’ attitudes, we find that more altruistic people are more in favor of migration than more selfish people and that center right–wing oriented subjects propose lower contribution levels than center left–wing oriented subjects. We conclude that economic motives are a crucial factor for accepting or rejecting welfare migration. Therefore, a key to promoting acceptance of new migrants is to ensure and then communicate that their net effect on growth, society, and the public purse is positive.

**Supplementary Information:**

The online version contains supplementary material available at 10.1007/s43546-022-00356-6.

## Introduction

Migration is, and has long been, a reality in Western societies. Flows of people migrating to other countries have never been larger in peace time and estimates show that they will double by 2050 (EPSC 2015). Today, approximately 37 million people born outside the European Union already reside in it, making up around 7% of its total population (Eurobarometer 2018). In the United States the respective numbers are 45 million people making up 14% of its population (CensusBureau 2016). On top of these numbers, there will be a need for migration in the future for developed countries due to declining birth rates and aging societies. The number of people of working age supporting each retiree over 65 continues to fall across the developed world. Twenty years ago, the worker/retiree ratio was 4:1 across the European Union. Today it is close to 3:1 and, even with current levels of migration, it is set to fall to 2:1 by 2050 (Eurostat, nd). Aging Western societies need migration in order to keep their social security systems and pensions safe for the future. Thus, a successful handling of migration appears to be one of the crucial challenges for the future of Western societies.^1^

In recent years, many Western societies have, however, witnessed a surge in anti-migrant sentiment, and a large proportion of the population views migration as one of the most pressing issues facing their country. In heated debates, facts are often ignored or misrepresented—for instance, 51% of Europeans (wrongly) believe that migrants do not contribute to taxes as much as they benefit from social services and welfare programs (Eurobarometer 2018). It is further argued that social welfare benefits serve as a driver for migration and have an effect on the qualification (or lack thereof) of attracted migrants (Razin and Wahba 2015). Political parties and politicians who have tapped into these concerns have gained support in the last few years, such as the “Front National” in France, the “Alternative für Deutschland” in Germany or Donald Trump in the United States. In fact, migration was a central issue in almost every national election in the European Union in the past few years.^2^

Despite the often negative media coverage and sentiment among people, evidence on migration and its benefits or detriments to receiving societies, respectively, is often mixed.^3^ Many developed countries, among others Australia, Canada, New Zealand, Singapore, and the United States, have built their prosperity on immigration, benefiting from cultural diversity and the entrepreneurial spirit of migrants. Recently, an OECD report estimated that migrants account for 47% of the increase in the workforce in the United States, and 70% in Europe over the past ten years (Liebig and Mo 2013). Usually, migrants do not negatively influence the public purse of the receiving countries. Some studies point out that the effects are marginal and countries like Switzerland or Luxembourg have even seen a net benefit of about 2% of GDP from migration, as migrants are usually young and have a long working life ahead of them (Bodvarsson and Van den Berg 2013; Card et al. 2012; OECD 2010; Ottaviano and Peri 2012). However, other studies report negative effects of migration on wages and employment of natives (Borjas 2003, 2014).^4^ The somewhat inconsistent results may in part be attributable to researchers approaching migration and its effects from very different perspectives (e.g., Borjas 1999; Riphahn et al. 2010; Zorlu 2013). The costs and benefits of welcoming immigrants have been investigated from a macroeconomic perspective by Tumen (2016) and from a market design perspective by Moraga and Rapoport (2014). Grigorieff et al. (2017) studied whether providing information about immigrants affects people’s attitudes towards them. From a theoretical perspective, Schultz and Sjöström (2001) developed a two-community model in which a district might experience congestion in the consumption of local public goods because it attracts new immigrants. As the mentioned studies take different research questions and use different models to answer them, we should not be surprised to also see different results and conclusions in this debate.

The present paper will not be able to resolve all disputes in this debate, but we add to the existing literature by exploring potential drivers behind welcoming or opposing migration in an economic experiment. This method allows a better control of variables and factors that are unobservable in real life. We set up a novel experimental design in which two societies exist in parallel, differing with respect to their level of welfare, i.e., there is a “rich” and a “poor” society/group. Subjects in each group interact in a uniform-contribution public good game for eight periods. In this version of the game, subjects decide in each period on the percentage of their periodic endowment they and all other society members should contribute to a public pool, which is then multiplied by a predefined factor and distributed equally among all group members. The actual contributions to be paid by all group members are set as the median of all proposed percentages within a group; a mechanism that is comparable to a perfectly enforced tax. Additionally, the experiment is designed to allow the migration of one member of the poorer society to the richer society. If an ordinary majority in the rich society supports a group change, one randomly chosen subject from the poorer society is allowed to change to the richer society. Within this framework, we attempt to address two distinct research questions (RQ).

### Research question 1

How does **subjects’ voting behavior** vary conditional on economic prospects, group assignment mechanism, (un)certainty regarding group membership, and personal characteristics?

### Research question 2

How do **individually proposed contributions, respectively their median,** to the public pool vary conditional on group assignment mechanism, (un)certainty regarding group membership, and personal characteristics?

With RQ 1, we aim to better understand subjects’ decision for or against migration while with RQ 2 we analyze subjects contributions to the public pool. Within each RQ, we analyze subjects’ behavior along different dimensions. First, motivated by an often cited argument in the migration debate, we are interested in how (potential) changes of the future economic situation after migration influences subjects’ decisions and behavior. Second, we use two group assignment mechanisms, i.e., subjects are either assigned randomly or through a real effort task to the rich and poor society, respectively. This condition is motivated by the observation that the extent to which someone is related to a group affects his or her behavior towards outsiders (Akerlof and Kranton 2000; Charness et al. 2007; Chen and Li 2009; Tajfel et al. 1971), and that perceived merit can influence distributional preferences (Burrows and Loomes 1994; Cappelen et al. 2013, 2017; Hoffman and Spitzer 1985). Third, motivated by the theory of Rawls (1971) concerning the veil of ignorance, we investigate how (un)certainty about group membership at the time of the vote for migration impacts the voting decision. Fourth, we investigate the impact of subjects’ personal characteristics on their voting behavior and the proposed contributions.

Our results suggest that (i) subjects mostly follow economic considerations in their decisions, i.e., most of the subjects vote in favor of a group change if they expect a higher individual payout and vote against a group change if it would decrease their payoff. This result highlights the role of material interest in accepting migration. Furthermore, we find that subjects’ individual attitudes significantly correlate with voting behavior, highlighting the role of such preferences in accepting migration. In particular, (ii) subjects with altruistic preferences are significantly more likely to vote in favor of a group change than subjects with more selfish preferences. Additionally, (iii) center right–wing oriented subjects propose lower contribution rates to the public good than center left–wing subjects. Manipulating the group assignment mechanism and the level of (un)certainty regarding subjects’ group assignment, we find minor variations, with the main conclusions from above remaining unchanged. An interesting side result for the public good researcher-community is that the uniform-contribution public good game yields high contribution rates with an average of 90%, hence, such a setup may make sense if one desires high contribution rates, e.g., in a baseline treatment.

## Experimental design

### Session sequence and overview

The experiment consists of two parts, as depicted in Table 1. Part 1 (see “Part 1: Equality equivalence test”) consists of a test eliciting subjects’ distributional preferences. In part 2 (see “Part 2: Uniform-contribution public good game with migration mechanism”), eight subjects form a cohort, which is then split into two groups, one (group high) with a higher endowment than the other (group low). Within each group, subjects play eight periods of a uniform-contribution public good game with the possibility of a group change after period 4, which might impact the economic prospect of that society. Within this environment, we implement treatment manipulations along two dimensions, studying a total of four treatment conditions. First, we examine whether different group assignment mechanisms have an impact on the likelihood to support or oppose migration. We do so by varying whether being in group high is due to pure luck, i.e., using a random assignment mechanism (rand), or due to “merit”, i.e., employing a real effort task (et). Second, we investigate how (un)certainty about subjects’ group membership at the moment of the vote about migration influences their voting behavior. Here, we implement one scenario in which subjects know their group membership (cer), while in the other they do not have this information at the time of the vote (unc). At the end of the experiment, subjects participate in a post–experiment questionnaire including questions regarding social ties (preferences concerning politics, their attitude towards migration, and their contact frequency with migrants; Eurobarometer 2018) and demographics (age, gender, education, mother tongue, and parents’ origin). After completing this questionnaire, subjects receive their payout and leave the lab. See Appendix A for experimental instructions and Appendix B for screenshots of the software.

**Table 1:** Session sequence and treatment overview

Part 1: Equality equivalence test	
Part 2: Uniform-contribution public good game with potential group change	
Real effort task in et	
Group assignment according to the scores in the real effort task (et) or random (rand)	
and voting stage (order conditional on treatment manipulation, i.e., cer or unc)	
Four periods of the uniform-contribution public good game	
Potential group change including multiplier change	
Four periods of the uniform-contribution public good game	
Post–experiment questionnaire	
Payout	

The table gives an overview of the sequence of the different decision stages in each treatment

### Part 1: Equality equivalence test

At the beginning of the session, we elicit subjects’ distributional preferences using the Equality Equivalence Test (eet) of Kerschbamer (2015). For the implementation, we used the software module of Holzmeister and Kerschbamer (2019). The eet asks subjects to make ten decisions. In each decision, the test presents subjects with two pairs of payouts in which each pair specifies a payout for another randomly matched player and one’s own payout, respectively. The ten decisions differ by the proportion of the own payout compared to the other subject’s payout. Based on the collected data, it is possible to characterize subjects according to one of nine archetypes (spiteful, kick-down, equality averse, envious, selfish, kiss-up, inequality averse, maximin, and altruistic). We will use this characterization later in the analysis to explain voting behavior on migration preferences and subjects’ proposed contributions. At the end of this part, subjects are paired and one decision situation is chosen for payout by a random mechanism. One of the two subjects receives the payout that results from her own decision while the other subject receives the related payout.

### Part 2: Uniform-contribution public good game with migration mechanism

In the main part of the experiment, eight subjects form one cohort, which is then split into two groups. In the paper, we refer to these groups as high and low providing an indicator for the endowment subjects receive within a group. In the experiment, however, we labeled these two groups neutrally as A and B, respectively, with labels being different for half of the sessions, to eliminate framing effects caused by group notations. Group high consists of five subjects, each receiving an endowment of 20 taler at the beginning of each period, while group low consists of three subjects each receiving an endowment of 10 taler. Endowments are reset at the beginning of each period.^5^ Several considerations determine the choice of group sizes. First, for group high, an uneven number of group members ensures clear majority votes in the migration decision (outlined later in this section). We decided to have five subjects to align with standard group sizes in the literature. Second, as the focus of the experiment is on the migration decisions of group high, we chose to have a lower number of members in group low for fairness concerns (a lower number of subjects in group low keeps the number of subjects that might be disappointed by a low payout within limits). Third, these parameter choices allow us to run a considerable number of independent cohorts, to optimally use the available lab capacity, and to keep costs within reasonable limits.

Subjects in each group interact in a repeated uniform-contribution public good game (uc-pgg) over eight periods. As indicated in Table 1, the eight periods are split into two blocks of four periods each. The framework of the experiments calls for this split so that there is one block of periods before and one after a potential migration. We decided to have four periods in each block to allow groups to converge to a certain behavior before and after the potential migration. At the beginning of each period, subjects in both groups anonymously propose a contribution level (to be paid by every member of the group) to the public pool. To increases comparability across the two groups, the contribution level is indicated in percent and can be chosen in the range 0–100% (in steps of 10 percentage points). The collected data on proposed contributions is used to determine a uniform contribution, which is binding for all group members, and it is computed as the median of all proposed percentages. For an even number of group members, the mean value of the two middle percentage values forms the median. Everyone in a group has to pay the same percentage of the endowment and it is neither possible to reduce the contribution (comparable to tax avoidance) nor can subjects add (voluntary) contributions.

In a next step, we determine subjects’ individual payout for the period. Following standard procedures for voluntary-contribution public good games (vc-pgg), the payout consists of two parts. First, subjects keep the part of their initial endowment that they did not contribute to the public pool. Second, we sum up the contributions by all group members and multiply this sum by a certain factor (initially 1.5). This amount is then redistributed to the members of the group in equal shares. Equation 1 summarizes the calculation of subject *i*’s period earnings.

1 $$\begin{aligned} \textsc {period earnings}_{i}= & \,{} \textsc {endowment}_i - \textsc {contribution}_i \nonumber \\&+ \frac{\sum _{i=1}^{N} \textsc {contributions}\cdot \textsc {factor}}{N} \end{aligned}$$

where *N* is the total number of subjects in a group. Note, that the period earnings of each subject are put into a separate account and are not carried over to the next period.^6^

The experiment is designed to (potentially) allow one subject to change from group low to group high during the course of the experiment. For this purpose, we implement a voting stage before the beginning of the uc-pgg in which subjects are asked whether they are in favor of a group change or not. If an ordinary majority favors a group change, one *randomly* chosen subject from group low changes to group high after period 4. The subject who was chosen to change to group high also receives 20 taler as period endowment in each of the remaining four periods.^7^^,^^8^ Providing the migrating subject with the same endowment as the resident group members, we assume that these persons can, without frictions and immediately, contribute to the production of the public good. This procedure resembles a situation after successful integration of the migrant into the new group.^9^ The group change may potentially cause a change in the multiplier, which is used to determine the payoff from the public pool (see equation 1). Initially, the multiplier is 1.5, but when a group change takes place, it can increase to 1.8, decrease to 1.2 or stay constant at 1.5 from periods 5 to 8 with equal probabilities of 1/3.^10^ A higher multiplier will, ceteris paribus, increase subjects’ period earnings, while for a decreasing multiplier the opposite holds.

For the elicitation of subjects’ preferences regarding a group change, we use a conditional voting method, i.e., for each potential factor (1.2, 1.5, and 1.8) subjects have to indicate their preferences in a separate voting. Hence, subjects vote in favor or against a group change conditional on the multipliers. In the process, we present the different multipliers in random order for each subject to avoid biases caused by the order of presentation. This design feature assumes an almost clarivoyant society in which the members of the society would know about the effects of migration and could condition their voting behavior on this knowledge. In reality, however, one cannot perfectly anticipate the effects of migration on societies and we, therefore, later vary this feature of our design by using unconditional voting for the migration vote (see “An alternative voting procedure”). To determine the realization of the multiplier at the beginning of period 5, we use a deck of three cards with each card representing one of the multipliers, respectively. One subject in each cohort randomly picks a card, which determines the multiplier.^11^ If no group change is preferred by group high, the multiplier and the group size remain unchanged.

### Treatment manipulations

Within this framework, the treatment conditions vary the realizations of two variables. First, we use two different group assignment mechanisms, i.e., subjects are either assigned randomly or through a real effort task to the rich and poor society, respectively. Second, we introduce (un)certainty about group membership at the time of the vote for migration. By manipulating two different factors with two realizations each, our study design is a 2x2 design, which implies a total of four different treatment conditions. Table 2 shows an overview of the conditions.

**Table 2:** Treatment overview

		(1) Group assignment	
		rand	et
(2) Group status	cer	$\textsc {cer\_rand}$	$\textsc {cer\_et}$
	unc	$\textsc {unc\_rand}$	$\textsc {unc\_et}$

cer/unc stands for certainty, respectively uncertainty; rand stands for random group assignment and et for group assignment based on the results of the effort task

The first treatment manipulation concerns the assignment of the eight subjects in a cohort to one of the two groups (high or low) at the beginning of the experiment. The manipulation is motivated by the observation that the extent to which someone is related to a group affects his or her behavior towards outsiders (Akerlof and Kranton 2000; Charness et al. 2007; Chen and Li 2009; Tajfel et al. 1971), and that perceived merit can influence distributional preferences (Hoffman and Spitzer 1985; Burrows and Loomes 1994; Cappelen et al. 2013, 2017). In particular, we implement two different group assignment mechanisms. Either subjects are randomly assigned to the groups (rand) or they are allocated according to their ranks in a real effort task (et).^12^ We determine these ranks by using a slider task in which subjects have two minutes to position as many sliders as possible on the value of 50 on a line ranging from 0 to 100 (Gill and Prowse 2012). In a practice round, subjects had the possibility to familiarize themselves with the mechanics of the task. In the actual task, subjects see 48 sliders on the screen. The five subjects with the highest number of correctly positioned sliders are assigned to group high while the other three subjects are assigned to group low. Subjects know in advance that the outcome of the slider task influences their group assignment. We chose the slider task because we want subjects to have equal opportunities to join group high, which are not depending on mathematical skills, educational background, or any other prior advantages. The idea is that subjects who are prepared to invest more effort in the task end up in group high. By varying the allocation mechanism, we aim to create a stronger feeling of entitlement among members of group high in et than in rand.

As a second treatment manipulation, we introduce certainty or uncertainty, respectively, regarding the group membership at the time of the vote, i.e., subjects either know that they are in group high or not at the time of the vote about welcoming or opposing migration. The idea for this variation is based on the theory of Rawls (1971) concerning the veil of ignorance. We suppose that the (un)certainty regarding subjects’ status within a cohort influences their decision regarding a group change. In cer, subjects already know their group membership when voting on the group change, i.e., subjects are assigned to group high or low and then only members of group high are allowed to vote. In contrast, in unc all subjects first vote on the group change before being assigned to the groups according to one of the two mechanisms (rand or et). Naturally, only votes of subjects who are later assigned to group high determine the decision on the group change.

### Conjectures

To guide the analysis of our research questions, we formulate behavioral conjectures capturing the different parts of the RQs. We present our expectations for RQ 1 (How does subjects’ voting behavior vary conditional on economic prospects, group assignment mechanism, (un)certainty regarding group membership, and personal characteristics?) in Conjectures 1.1 to 1.6.

For the first part of RQ 1, we assume that subjects consider their financial interests when making a decision in favor of or against migration. Thus, we expect subjects to largely oppose migration when this would lead to a decrease in the multiplier while we expect high approval rates for increases in the multiplier. If the multiplier remains unchanged, we expect approval rates to be between the two more extreme realizations without having a clear prior whether the majority would prefer migration or not. We formulate the following conjecture:

#### Conjecture 1.1

The average share of subjects voting in favor of migration increases in the multiplier.

The second part of RQ 1 studies the impact of the first treatment manipulation on voting behavior. In contrast to a random group assignment (rand), by using a real effort task for the group assignment (et), we aim to create a feeling of entitlement within the rich group. With a higher feeling of entitlement, we suspect that subjects are less in favor of migration and migration votes decrease (see e.g., Hoffman et al. 1994; Frohlich et al. 2004; Jakiela 2011; Cappelen et al. 2013; Jakiela 2015). We formulate the following conjecture:

#### Conjecture 1.2

The average share of subjects voting in favor of migration is lower in et than in rand.

The third part of RQ 1 covers the impact of the level of (un)certainty regarding subjects’ group assignment at the time of the migration vote on voting behavior. Either subjects already know their group membership when voting on the group change (cer), or subjects first vote on whether to allow a group change before being assigned to the groups (unc). Here, we conjecture that subjects in the treatment condition unc vote more often in favor of a group change than subjects in the treatment condition cer due to the uncertainty regarding their group assignment at the time of the vote. Not knowing precisely their membership in group high or low, subjects might aim at increasing their chances of ending up in group high at least for the second part of the experiment. We formulate the following conjecture:

#### Conjecture 1.3

The average share of subjects voting in favor of migration is higher in unc than in cer.

The fourth part of RQ 1 explores how personal characteristics and convictions influence subjects’ voting decision. We formulate three conjectures based on the following considerations. First, in recent years, many Western societies have witnessed a surge in anti-migrant sentiment with particularly right–wing parties tapping these concerns (Werts et al. 2013; Shehaj et al. 2021). Based on these observations, we would expect subjects who identify themselves as rather right–wing to be less in favor of migration than subjects identifying themselves as rather left–wing. Second, there is ample research studying the influence of altruism on voting behavior (see, e.g., Andreoni (1990) and Croson (2007) and the literature citing them), which strongly suggests that altruism will be positively related with the readiness to accept new subjects to the group. Third, we touch on potential gender effects in voting behavior. We formulate the following conjectures:

#### Conjecture 1.4

The average share of subjects voting in favor of migration is lower for subjects who locate themselves more on the right–wing political spectrum.

#### Conjecture 1.5

The average share of subjects voting in favor of migration is higher for more altruistic subjects.

#### Conjecture 1.6

A person’s gender does not impact the voting in favor of migration.

We present our expectations for RQ 2 (How do **proposed contributions to the public pool and their median** vary conditional on group assignment mechanism, (un)certainty regarding group membership, and personal characteristics?) in Conjectures 2.1 to 2.6.

Before we analyze the different aspects of RQ 2, we study the overall contribution levels. As outlined above, we use a modified pgg without the possibility to free-ride, thus changing the equilibrium predictions relative to a standard vc-pgg. As it is in the economic interest of everybody to maximize contributions, and as these are perfectly enforced, we expect contributions close to 100%. We formulate the following conjecture:

#### Conjecture 2.1

A common commitment mechanism results in high and constant contributions.

The first part of RQ 2 studies the impact of the group assignment mechanism on subjects’ contributions. Kesternich et al. (2018) report differences in contributions when group assignment is done by a random vs. an effort-based mechanism, and especially in the latter the feeling of entitlement should be more prevalent. However, as we have perfect enforceability of the contribution level, irrespective of group assignment mechanism, going for maximal contributions is always the dominant strategy and we thus expect no differences in contributions conditional on the group assignment mechanism.We formulate the following conjecture:

#### Conjecture 2.2

The group assignment mechanism does not influence the level of contributions to the public pool.

Turning to (un)certainty about group membership at the time of the vote for migration and its potential influence on contribution levels, we follow the same line of reasoning as above: irrespective of the level of (un)certainty individual payouts of everybody are maximized if contributions levels are maximized, and as these are perfectly enforced, we again expect high and unchanging contribution levels. We formulate the following conjecture:

#### Conjecture 2.3

The level of (un)certainty regarding the group assignment does not impact contributions to the public pool.

Turning to gender and its potential influence on proposed contribution levels, the literature does not deliver a clear picture. For example, Brown-Kruse and Hummels (1993) report higher contributions for male than for female subjects while Cadsby and Maynes (1998) find the opposite effect. Some report very small gender effects, which sometimes even vanish for repeated games (e.g., Chaudhuri (2011); see Ledyard (1995) for a discussion). We formulate the following conjecture:

#### Conjecture 2.4

A person’s gender does not influence the proposed contributions to the public pool.

As written above, people self-identifying as more right-wing are less ready to contributing to public goods, and thus we expect them to deviate from the optimum of 100% contributions and to propose lower contribution levels to the public good. We formulate the following conjecture:

#### Conjecture 2.5

More right–wing oriented subjects propose significantly lower contribution levels than more left–wing oriented subjects.

Finally, on altruistic attitudes, the literature on pgg finds that more altruistic people contribute more in vc-games (see Andreoni (1990) and Croson (2007) and the literature citing them). As we have a modified pgg in which proposing 100% contributions anyways, we do not expect a significant effect of altruistic attitudes on contributions levels. We formulate the following conjecture:

#### Conjecture 2.6

There is no influence of a subjects’ altruistic attitude on proposed contributions to the public pool.

### Implementation

The sessions of the experiment were conducted in May, June, July, and December 2019 at the EconLab of the University of Innsbruck with a total of 384 students. In each of the four treatments, we collected data from 12 cohorts and each cohort consisted of eight participants. On average, subjects were 23 years old and 55% of them were female. See Table C.1 in Appendix C for more details. Subjects were recruited using hroot (Bock et al. 2014) and the experiment software was programmed with oTree (Chen et al. 2016). At the beginning of each session, subjects had time to individually read the instructions for part 1 (eet) and ask questions, which were answered privately. Then, we handed out the instructions for part 2 and subjects had time to individually read them and ask questions before the experiment started. Across all treatments, subjects earned on average € 19.^13^

## Results

We organize the results in three sections. “RQ 1: Voting behavior on migration” and “RQ 2: Proposed and median contribution levels” provide the answers RQ 1 and RQ 2, respectively. Within each of these sections, we organize the results using the same numbering as used with the conjectures formulated in “Conjectures”, i.e., Result 1.1 relates to Conjecture 1.1. “An alternative voting procedure” presents two additional treatments that modify the voting mechanism.

### RQ 1: Voting behavior on migration

**Fig. 1:**
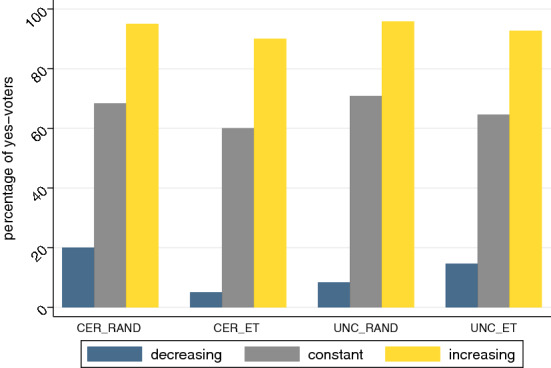
Average share of subjects voting in favor of a group change across treatments. The graph represents percentage proportions of subjects voting in favor of a group change across all treatments. Percentage proportions of yes-voters are depicted for the decreasing multiplier scenario (i.e., multiplier 1.2, blue bar), the constant multiplier scenario (i.e., multiplier 1.5, gray bar) and the increasing multiplier scenario (i.e., multiplier 1.8, yellow bar)

Figure 1 presents the average share of subjects voting in favor of a group change in each treatment considering all collected votes. The blue bars represent percentage shares of yes-votes in case the multiplier changes to 1.2 after a group change (i.e., a decreasing multiplier), gray bars give the percentage of yes-voters in case the multiplier stays at 1.5 (i.e., a constant multiplier), and the yellow bars show the respective percentage of yes-votes in case the multiplier changes to 1.8 (i.e., an increasing multiplier).

#### Result 1.1

The average share of subjects voting in favor of migration increases in the multiplier.

**Support.** We observe a uniform pattern in the preference for migration in all treatments indicating an increasing approval rate with increasing multipliers. Migration approval rates are less than 20% across all treatments in case the multiplier decreases to 1.2 after a group change, while in the increasing multiplier case approval rates are 90% and above. When the multiplier remains constant (i.e., multiplier 1.5) a clear majority of between 60% and 71% of subjects vote in favor of accepting a new group member to group high.^14^ Using pooled data from all treatments, migration approval rates are 12% for a declining, 66% for a constant, and 94% for an increasing multiplier. When testing for differences conditional on the multiplier, we find significant differences in all possible comparisons using two-sided two-sample test of proportions.^15^ We interpret this pattern as an indication that subjects act ‘rational’, i.e., in their economic interest, when deciding about a group change. Approval rates are very low when it is disadvantageous for their payoff but very high when subjects benefit from the group change. Hence, their own economic consequences are a major driver of subject’s voting behavior. The individual voting preferences are also reflected in actual outcomes when only the votes of group high are considered. In particular, in 13 of the 16 cohorts with a constant multiplier after a group change, migration was allowed. Migration was also approved in all 16 cohorts in which the multiplier increased to 1.8, while none of the groups in which the multiplier would have decreased to 1.2 allowed migration.

Analyzing the data in more detail, we find several significant treatment difference of subjects’ voting behavior in the decreasing multiplier case: subjects’ approval is highest (at 20%) in treatment $\textsc {cer\_rand}$. In particular, using two-sided two-sample test of proportions, we find significant differences between treatment $\textsc {cer\_rand}$ and $\textsc {cer\_et}$ ($z = 2.48$, $p =.0130$) as well as $\textsc {cer\_rand}$ and $\textsc {unc\_rand}$ ($z = 2.20$, $p =.0281$). The difference between $\textsc {cer\_rand}$ and $\textsc {cer\_et}$ points to an ‘entitlement effect’, as those who did well in the real effort task were less willing to accept a new group member, who obviously did worse in the slider task.^16^ We find that female subjects solve on average more than three sliders less than male subjects (*p* < 0.001%; similar results are reported by Lezzi et al. (2015) and Murad et al. (2019)). Furthermore, subjects’ who believe to be assigned to group high with higher probability solve significantly more sliders in the real effort task, indicating that they were able to predict their own performance to some degree (*p* < 0.001%). The results are the same when we add data from the additional et treatment presented in “An alternative voting procedure” (model 2). Note that in treatment $\textsc {cer\_et}$, the proportions in favor of accepting a new group member are also the lowest across all four treatments for each of the three multipliers (i.e., 1.2, 1.5, and 1.8). Hence, subjects who performed well in the slider task and know their group allocation at the time of the vote are less likely to vote in favor of a group change than subjects who are randomly allocated to group high. The second significant difference ($\textsc {cer\_rand}$ and $\textsc {unc\_rand}$) suggests that if subjects are certain about their group membership and are randomly assigned (i.e., they were lucky to get into group high), they are more likely to vote in favor of allowing one more subject to join this group than subjects who are still uncertain about their group affiliation at the time of the vote.

To analyze subjects’ voting behavior in more detail, we run regressions relating voting behavior to treatment conditions and subjects’ individual characteristics elicited in the eet and the post-experiment questionnaire. In Table 3, we present the results of logistic regressions, with clustered standard errors on the cohort level and $\textsc {vote\_dec}$ (model 1), $\textsc {vote\_const}$ (model 2), and $\textsc {vote\_inc}$ (model 3) as dependent variables. These are dummy variables where 1 means a subject voted in favor of a group change and 0 indicates a vote against a group change. dec, const, and inc represent the multipliers 1.2, 1.5, and 1.8, respectively. The set of independent variables consist of three dummies marking treatment manipulations ($\textsc {et}$, $\textsc {cer}$, and their interaction $\textsc {et x cer}$) and of three variables capturing subject characteristics ($\textsc {female}$ for gender, $\textsc {pol\_preference}$ proxying for political preferences with higher values indicating right–wing preferences, and $\textsc {altruistic-like}$ to index altruistic-like subjects).^17^ Additionally, we interact the treatment dummies with the three variables on subject characteristics to identify potential treatment specific effects of these variables on voting behavior.

#### Result 1.2

The group assignment mechanisms does not influence the average share of subjects voting in favor of migration.

#### Result 1.3

The level of (un)certainty regarding the group assignment does not influence the average share of subjects voting in favor of migration.

**Support.** We find that neither of the treatment conditions, i.e., $\textsc {et}$ and $\textsc {cer}$, are significantly related to voting behavior. However, consistent with the observation in Fig. 1, a treatment effect emerges for the decreasing multiplier scenario between $\textsc {cer\_rand}$ and $\textsc {cer\_et}$. In particular, the interaction effect $\textsc {et x cer}$ is significant at the 1% level. Thus, subjects seem to value being in group high more if they achieved being in this group through their own effort (i.e., group allocation as a result of scores in the slider task) than if they were assigned randomly. As a consequence, in $\textsc {cer\_et}$, members of group high are less likely to vote in favor of accepting a new group member. We do not find treatment effects for the variation of the group assignment (i.e., et vs. rand) for the remaining treatments (i.e., models 2 and 3). We thus conclude that earned entitlement owing to effort does not in all situations impact subjects’ voting behavior in our setting.^18^ Furthermore, there is no clear evidence that subjects’ (un)certainty regarding their group assignment (i.e., cer vs. unc) impacts their voting behavior.^19^

#### Result 1.4

A person’s gender does not impact the voting in favor of migration.

#### Result 1.5

Subjects’ political preferences do not significantly impact their voting behavior in favor of migration.

#### Result 1.6

The average share of subjects voting in favor of migration is higher for more altruistic subjects.

**Support.** Concerning the impact of subjects’ individual characteristics on voting behavior, our result do not reveal any significant impact of gender on voting behavior. In contrast with our conjecture, political preferences only have a marginal impact on subjects’ voting behavior, in particular in condition cer. Here, we find that subjects who know about their group assignment and lean more towards the right–wing political spectrum are less supportive of migration even when migration would result in an increasing multiplier (Werts et al. 2013; Shehaj et al. 2021). In contrast, subjects with more altruistic-like preferences are more likely to vote in favor of a group change than subjects with selfish-like preferences. While the coefficient values are positive for in all estimated models, they are significant only in the constant multiplier case (*p* < 0.05). These findings are, e.g., in line with results presented in Andreoni (1990) and Croson (2007), which strongly suggest that altruism is positively related with the readiness to accept new subjects to the group. In sum, subjects’ preferences regarding a group change do not strongly depend on how groups are created, or whether group identity is known, but rather on their altruistic preferences as well as on their expectation of whether accepting a new group member would have positive or negative economic consequences for them.

**Table 3:** Logistic regressions explaining subjects’ voting behavior in favor or against a group change

	$\textsc {vote\_dec}$	$\textsc {vote\_const}$	$\textsc {vote\_inc}$
	(1)	(2)	(3)
$\textsc {et}$	0.936 (1.180)	− 0.326 (0.814)	1.118 (1.422)
$\textsc {cer}$	0.758 (1.369)	0.854 (0.851)	2.203 (1.655)
$\textsc {et}$ $\times$ $\textsc {cer}$	− 1.946** (0.713)	0.135 (0.471)	0.012 (0.923)
$\textsc {female}$	0.756 (0.894)	0.427 (0.323)	0.153 (0.829)
$\textsc {et}$ $\times$ $\textsc {female}$	− 0.411 (0.894)	− 0.188 (0.453)	− 1.164 (0.947)
$\textsc {cer}$ $\times$ $\textsc {female}$	− 1.378 (0.976)	− 0.187 (0.462)	− 0.584 (0.982)
$\textsc {pol\_preference}$	− 0.385 (0.252)	− 0.099 (0.189)	0.586 (0.358)
$\textsc {et}$ $\times$ $\textsc {pol\_preference}$	− 0.281 (0.315)	0.073 (0.222)	− 0.508 (0.385)
$\textsc {cer}$ $\times$ $\textsc {pol\_preference}$	0.489 (0.346)	− 0.420 (0.215)	− 0.892* (0.383)
$\textsc {altruistic-like}$	1.532 (0.956)	0.820* (0.379)	0.697 (0.869)
$\textsc {et}$ $\times$ $\textsc {altruistic-like}$	0.660 (1.097)	− 0.173 (0.509)	0.261 (1.029)
$\textsc {cer}$ $\times$ $\textsc {altruistic-like}$	− 0.070 (1.033)	− 0.363 (0.559)	− 0.826 (1.007)
Constant	− 3.095** (1.116)	0.594 (0.671)	1.645 (1.037)
Observations	307	307	307
Pseudo R$^{2}$	0.169	0.052	0.076
Chi$^{2}$	49.552	31.943	18.657

The table shows estimates from logistic regressions with $$\textsc {vote\_dec}$$ (model 1), $$\textsc {vote\_const}$$ (model 2), and $$\textsc {vote\_inc}$$ (model 3) as dependent variables. All three variables are dummy variables, which take on a value of 0 if subjects vote against a group change and 1 if subjects vote in favor of a group change. $$\textsc {dec}$$, $$\textsc {const}$$ and $$\textsc {inc}$$ represent the conditions of a decreasing (1.2), constant (1.5) and increasing (1.8) multiplier. $$\textsc {et}$$ and $$\textsc {cer}$$ are dummies for the treatment manipulations. $$\textsc {et}$$ takes on a value of 1 if subjects were assigned to groups by conducting the slider task or 0 if they were randomly assigned. $$\textsc {cer}$$ is 1 if the vote regarding the group change is conducted after the group assignment and 0 if it takes place before the group assignment. $$\textsc {et x cer}$$ is the interaction term for $$\textsc {et}$$ and $$\textsc {cer}$$ and captures the marginal effect. The independent variable $$\textsc {female}$$ is a dummy variable and takes on values of 0 for male subjects and 1 for female subjects. $$\textsc {pol\_preference}$$ is an ordinal variable from 0 to 6 where 0 means a subject self-identified as rather left–wing, while 6 means a subject self-identified as rather right–wing. $$\textsc {altruistic-like}$$ is a dummy variable taking on values of 0 if subjects’ distributional preferences are categorized either as spiteful, kick-down, equality averse, envious or selfish and 1 if their distributional preferences are either kiss-up, inequality averse, maximin or altruistic, respectively. Additionally, the regressions contain terms interacting the treatment dummies with the three variables on subject characteristics. Clustered standard errors on cohort level are in parentheses. $$^*$$, $$^{**}$$, and $$^{***}$$ represent the 5%, 1%, and 0.1% significance level, respectively

### RQ 2: proposed and median contribution levels

After having analyzed subjects’ voting behavior, we now examine median contribution levels in the uc-pgg and subjects’ individually proposed contribution levels to provide answers to the different dimensions of RQ 2.

#### Result 2.1

A common commitment mechanism results in high and constant contributions.

**Fig. 2:**
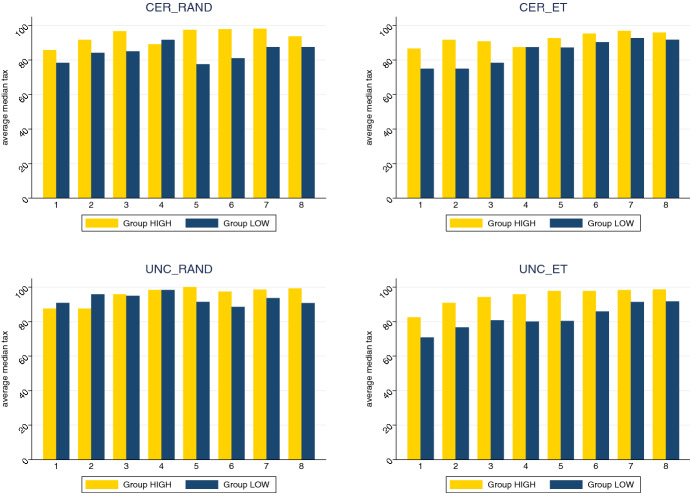
Average median contribution levels for each period, treatment and group. The graph shows average median contribution level for each treatment over all eight periods, separated by group high (yellow bars) and low (blue bars)

**Support.** Figure 2 indicates that median contribution levels are markedly higher compared to standard public good games with a voluntary contribution mechanism. On average, median contribution levels are almost always above 80%, while in a standard vc-pgg contributions on average do not exceed 50%. Moreover, contributions usually decrease over time, while they are stable or even increasing in our experiment, particularly in group high.^20^ These results are comparable to the results of uniform common commitment mechanisms as discussed, e.g., in Gallier et al. (2016), Kesternich et al. (2018), and Schmidt and Ockenfels (2021). Hence, a mandatory contribution level determined by aggregating group preferences seems to be a suitable instrument to ensure high and stable contribution rates in a public good game due to the absence of free-riding opportunities (see also Huber et al. (2018) for a public good game with contribution levels set by vote). We even find that average median contribution levels of periods 5 to 8 are significantly higher than the levels of periods 1 to 4 (Wilcoxon rank-sum test for all treatments with unconditional voting; $z = 3.254$, $p = 0.0011$).^21^ Most likely, participants increasingly understand that they maximize their payoffs the higher the contribution levels due to the absence of free-riding possibilities in this setting. In contrast, standard public good games report decreasing contributions in repeated games (e.g., Isaac et al. 1984; Andreoni 1995; Fehr and Gächter 2000; Bochet et al. 2006). By visual inspection of Fig. 2, we additionally see that average contribution rates are higher in group high compared to group low over all treatments particularly from periods 5 to 8 (period 5: $z = 3.699$, $p = 0.0001$; period 6: $z = 3.096$, $p = 0.0020$; period 7: $z = 2.986$, $p = 0.0028$; and period 8: $z = 3.078$, $p = 0.0021$).

We continue our analysis by examining the effect of a *group change* on subjects’ proposed contribution levels. Overall, we find that subjects’ preferences regarding contribution levels in the uc-pgg are not significantly affected by a group change. First, we investigate how average proposed contribution levels after a potential group change (i.e., from periods 5 to 8) vary in case of a group change. For these cases, we neither observe a significant difference in group high between change and no-change cohorts ($z = 1.029$, $p = 0.3033$) nor do we find a significant difference between groups high and low in change cohorts ($z = 0.187$, $p = 0.8514$). Second, we examine the difference in proposed contribution levels between periods 4 and 5, i.e., where a potential group change might occur. Again, we do not find substantial differences between change and no-change cohorts in group high ($z = 0.745$, $p = 0.4561$) and we do not observe significant differences between groups high and low in change cohorts ($z = 0.287$, $p = 0.7743$).^22^ In order to obtain a clear understanding of the effects of a group change on subjects’ contribution preferences, we further look at the proposed contribution level of the group changer, i.e., the subject who was by chance chosen to change from group low to group high. We do not find any evidence for a deviating behavior of these subjects. Group changers do not contribute significantly less in period 5 compared to their members of group high ($z = 0.432$, $p = 0.6661$). They also do not propose significantly less in period 5 compared to period 4 ($z = 0.475$, $p = 0.6349$).

Having analyzed the effect of a group change on subjects’ contributions in the uc-pgg, we now examine the impact of the treatment conditions and individual characteristics on proposed contribution levels. Table 4 shows a GLS random effects regression with $\textsc {proposed contributions}$ as the dependent variable over all eight periods. Standard errors are clustered at the cohort level. The set of independent variables consist of two dummies marking treatment manipulations ($\textsc {et}$ and $\textsc {cer}$), of three variables capturing subject characteristics ($\textsc {female}$ for gender, $\textsc {pol\_preference}$ proxying for political preferences, and $\textsc {altruistic-like}$ to index altruistic-like subjects), and of $\textsc {period}$, which is a variable from 1 to 8 denoting the respective period in the uc-pgg. Additionally, the regression contains terms interacting the treatment dummies with the three variables on subject characteristics.^23^

#### Result 2.2

The group assignment mechanism does not influence the level of contributions to the public pool.

#### Result 2.3

The level of (un)certainty regarding the group assignment does not impact contributions to the public pool.

**Support.** We do not report any treatment effects regarding the group allocation mechanism, i.e., rand vs. et and the (un)certainty of the group assignment, i.e., cer vs. unc (see also Table C.3, Appendix C). The first result is in contrast with Kesternich et al. (2018) who report that a random group allocation limits efficiency gains if contributions are rule-based. Consistent to the visual inspection of Fig.  2 in which we show average median contribution levels in each period, we report a strong period effect regarding subjects’ proposed contribution levels. Specifically, as seen in Table 4, proposed contribution levels significantly increase from period 1 to 8.

**Table 4:** GLS random effects regression explaining individually proposed contribution levels over all eight periods

	$$\textsc {proposed contributions}$$
$\textsc {et}$	− 2.682 (5.867)
$\textsc {cer}$	− 5.995 (6.363)
$\textsc {et}$ $\times$ $\textsc {cer}$	7.276 (3.808)
$\textsc {female}$	− 7.665* (3.501)
$\textsc {et}$ $\times$ $\textsc {female}$	− 6.111 (3.896)
$\textsc {cer}$ $\times$ $\textsc {female}$	2.735 (3.965)
$\textsc {pol\_preference}$	− 3.886* (1.582)
$\textsc {et}$ $\times$ $\textsc {pol\_preference}$	0.761 (1.834)
$\textsc {cer}$ $\times$ $\textsc {pol\_preference}$	− 1.644 (1.838)
$\textsc {altruistic-like}$	− 3.212 (3.094)
$\textsc {et}$ $\times$ $\textsc {altruistic-like}$	− 3.096 (4.065)
$\textsc {cer}$ $\times$ $\textsc {altruistic-like}$	3.611 (4.012)
$\textsc {period}$	1.468*** (0.217)
Constant	95.113*** (5.366)
Observations	3008
R$^{2}: overall$	0.070
R$^{2}: within$	0.041
R$^{2}: between$	0.086

The table shows estimates from a GLS random effects panel regression with $$\textsc {proposed contributions}$$ as the dependent variable, which represents subjects’ proposed contributions in the public good game. $$\textsc {et}$$ and $$\textsc {cer}$$ are dummies for the treatment manipulations. $$\textsc {et}$$ takes on a value of 1 if subjects were assigned to groups by conducting the slider task or 0 if they were randomly assigned. $$\textsc {cer}$$ is 1 if the vote regarding the group change is conducted after the group assignment and 0 if it takes place before the group assignment. $$\textsc {female}$$ is a dummy variable and takes on values of 0 for male subjects and 1 for female subjects. $$\textsc {pol\_preference}$$ is an ordinal variable from 0 to 6 where 0 means a subject self-identified as rather left–wing, while 6 means a subject self-identified as rather right–wing. $$\textsc {altruistic-like}$$ is a dummy variable taking on values of 0 if subjects’ distributional preferences are categorized either as spiteful, kick-down, equality averse, envious or selfish and 1 if their distributional preferences are either kiss-up, inequality averse, maximin or altruistic, respectively. Additionally, the regression contains terms interacting the treatment dummies with the three variables on subject characteristics. $$\textsc {period}$$ is a variable from 1 to 8 and denotes the respective period in the uc-pgg. Clustered standard errors on cohort level (i.e., each cohort consists of eight subjects and group $$\textsc {a}$$ and $$\textsc {b}$$.) are in parentheses. $$^*$$, $$^{**}$$, and $$^{***}$$ represent the 5%, 1%, and 0.1% significance level, respectively

#### Result 2.4

Female subjects propose significantly lower contribution levels to the public pool.

#### Result 2.5

More right–wing oriented subjects propose significantly lower contribution levels than more left–wing oriented subjects.

#### Result 2.6

There is no influence of a subjects’ altruistic attitude on proposed contributions to the public pool.

**Support.** The regression results reveal that female subjects propose to contribute significantly less to the common pool than male subjects. Intuitively, this result differs from our expectation of the effect to occur in the opposite direction. However, existing literature on gender differences in standard public good games does not unambiguously agree on whether men or women contribute more to a common good. For example, Brown-Kruse and Hummels (1993) report higher contributions for male than for female subjects while Cadsby and Maynes (1998) find the opposite effect. Some report very small gender effects, which sometimes even vanish for repeated games (e.g., Chaudhuri (2011); see Ledyard (1995) for a discussion).^24^

In line with our conjecture, the results reveal a significant effect of subjects’ political preferences on their proposed contribution levels. In particular, more right–wing oriented subjects propose significantly lower contribution levels than more left–wing oriented subjects. Finally, we do not observe an influence of subjects’ altruistic attitude on proposed contributions to the public pool. For some readers results 2.5 and 2.6 may seem slightly contradictory, as there is literature suggesting a link between political orientation (or left- or right-wing preferences in elections) and personality traits like e.g., altruistic behavior. Often, people with altruistic personality traits (e.g., honesty-humility, agreeableness) are reported to favor left-wing oriented parties (see, e.g., Chirumbolo and Leone 2010 and Rooduijn et al. 2017 although some contradicting evidence is also reported (see, e.g., Wang 2016).) Note, however, that these studies investigate how altruism influences left- or right-wing preferences in elections whereas we investigate how altruism and political preferences influence contributions in our game. Therefore, the results are not perfectly comparable and it is difficult to derive implications from existing literature on this study.

### An alternative voting procedure

In this section, we present an extension of the experimental design, which augments the results related to subjects’ approval rates of migration. In this extension, we implement a different voting procedure determining whether a group change occurs or not. In particular, subjects now indicate their preferences regarding a group change of one member of group low to group high via *unconditional voting*, i.e., subjects decide whether to allow a group change or not in one vote rather than in individual votes for each of the three economic scenarios (i.e., multiplier 1.2, 1.5, and 1.8). Hence, at the time of the vote, subjects face a higher degree of uncertainty about the future multiplier in case of a group change because they solely know the probability of occurrence, which is 1/3 for each scenario. With this feature, the experimental design more closely reflects one of the main problems of migration policy. In reality, one cannot perfectly anticipate the effects the migration of individuals or groups has on societies.

In order to determine the realization of the multiplier at the beginning of period 5, we again use a deck of three cards, i.e., one card for each multiplier. One subject in a session, which consists of three cohorts, randomly picks one card. The drawn multiplier then applies for each of the three cohorts, which increases the uncertainty about the multiplier outcome in case of a group change compared to the situation when conditional voting is used. Similar to the basic experiment in which conditional voting was used, the multiplier and the group size do not change, if an ordinary majority of group high voted against a group change. Due to money and subject pool restrictions, we applied this alternative voting procedure only on two treatments, namely $\textsc {cer\_rand}$ and $\textsc {cer\_et}$. With this voting procedure, we ran 12 cohorts of each treatment with an additional 192 subjects who were again students from the University of Innsbruck with similar age and gender-ratio compared to the sample of the main experiment. Moreover, they received a similar average payout than subjects participating in the main experiment. See Table C.1 in Appendix C for more details.

#### Result 3.1

When unconditional voting is used, subjects who classify themselves as right–wing oriented voters are significantly less likely to vote in favor of a group change than left–wing oriented subjects.

**Support.** Regarding subjects’ voting behavior (RQ 1), we report approval rates in favor of a group change around 60%, which do not vary between treatments $\textsc {cer\_rand}$ and $\textsc {cer\_et}$ (two-sided two-sample test of proportions, $z = 0.56$, $p =.5753$). These approval rates are similar to the approval rates in the constant multiplier case (i.e., multiplier 1.5) presented in “RQ 1: Voting behavior on migration”. We further observe that in both treatments, migration was allowed in 15 of the 24 cohorts. Hence, as can be seen in three regressions models presented in Table C.5 of Appendix C, we do not report treatment differences regarding subjects’ voting behavior when using the unconditional voting for the migration vote. Like in the main experiment, we also link the voting behavior to subjects’ individual characteristics (see model 2 of Table C,5, Appendix C). Here, we observe that subjects who classify themselves as right–wing oriented voters are significantly less likely to vote in favor of a group change than left–wing oriented subjects. This finding is in contrast to results of the main experiment presented in “RQ 1: Voting behavior on migration”, where we do not find a significant effect of subjects’ decision to vote in favor of migration and their political preference. One explanation for this pattern might be that right–wing oriented voters dislike the higher degree of uncertainty being associated with unconditional voting and thus prefer to avoid uncertainty by voting against migration.

Based on these observations, we conclude that inducing a higher level of uncertainty to the voting procedure by applying unconditional voting yields very similar migration approval rates than in the constant multiplier case of the basic experiment. Additionally, we do not find any treatment effects. Thus, higher uncertainty leads to similar behavior than if subjects’ payoff is not affected by migration.

#### Result 3.2

When unconditional voting is used, female subjects propose significantly lower contributions to the public pool.

**Support.** Analyzing subjects’ proposed contributions, we follow a similar approach as outlined in “RQ 2: Proposed and median contribution levels” by running GLS random effects regressions. The results are in shown Table C.3. Models 2 (featuring interaction terms between the treatment dummy and the subjects’ characteristics) and 3 (adding additional control variables) in this table analyze data from the two treatments with unconditional voting. All results (apart from political preferences) remain unchanged. The coefficient for political preferences, significantly negative on the 5% level in model 1, remains negative but is no longer significant in models 2 and 3.

## Conclusion

By means of a controlled lab experiment, we explored several potential drivers for welcoming or opposing migration. In our experiment two societies (one “rich”, one “poor”) existed in parallel, and after several periods it was possible for a member of the poor society to migrate to the rich society – but only if the majority of voters in the rich society agreed to that. Within this setting, we explored the influence of different group assignment mechanisms (by merit or random) and tested whether (un)certainty of group membership at the moment of the vote about migration influenced the voting behavior. Our results suggest that (i) most subjects followed economic (rational) considerations in their decisions, i.e., they mostly voted in favor of a group change if they expected a higher individual payout and voted against a group change if it would decrease their payoff. This result highlights the role of material interest in accepting migration. Furthermore, we found that subjects’ attitudes significantly influence their voting behavior, as (ii) subjects with altruistic preferences were significantly more likely to vote in favor of a group change than subjects with more selfish preferences. With contributions in a modified version of the public good game set as the median of all proposed contribution rates, which were then paid by everybody, we found very high and increasing contribution rates with an average of 90%. However, (iii) center right–wing oriented subjects proposed lower contribution levels in this game than center left–wing subjects. Results (ii) and (iii), in contrast to (i), highlight the role of social preferences in accepting migration and in contributing to a public pool. From these results, we conclude that one key to promote acceptance of new migrants is to demonstrate that their net effect on growth, society, and the public purse is positive.

When interpreting these results, one has to consider several limitations. First, the focus was on welfare migration, i.e., people migrating from poor to rich societies mainly because of economic reasons or better prospects. Therefore, the study only captures a share of the worldwide migration and does not consider the type of migration when people are forced to migrate because of war or other conflicts. Second, the treatments in this paper do not consider significant issues that often drive the political discussion on welcoming or opposing migration. In particular, we do not study the impact on voting behavior that might emerge due to cultural (often religion, race, etc.) and/or educational differences between the two groups. An investigation into these issues might be an interesting endeavor for future research as, in addition to economic differences, often cultural or educational differences characterize the two regions involved in migration movements.

The setting proposed in this study is flexible enough to serve as a test bed for investigating the impact of other factors or migration polices on migration tendencies. To name one example, in the study, the migrating person was chosen randomly. Such a scheme is comparable to the green card in the US but countries use many other schemes as well. Therefore, one could think of other mechanisms like contribution-based mechanisms, application formats, or a point system, among others. Another example might be the variation of endowments. Our setting uses homogeneous endowments that only vary between groups. Here, future research could implement (systematic) variations to test how different endowment levels within a group would influence subjects’ preferences for migration. Moreover, one could implement real-effort tasks generating heterogeneity in endowments to see how this change influences the results compared to homogeneous endowments distributed without effort involved.

## Supplementary Information

Below is the link to the electronic supplementary material.

Supplementary file1 (PDF 602 KB)

## Data Availability

The data sets generated during and/or analysed during the current study are available from the corresponding author on reasonable request. We hereby declare that this paper reports all experimental sessions and treatments conducted within the course of this study.

## Footnotes

1 At the United Nations conference on December 10–11, 2018 in Marrakesh, Morocco, more than 150 states agreed on the Global Compact for Safe, Orderly and Regular Migration. The Global Compact for Migration is the first-ever UN global agreement on a common approach to international migration in all its dimensions. It is rooted in values of state sovereignty, responsibility-sharing, non-discrimination, and human rights, and emphasizes that a cooperative approach is necessary to optimize the overall benefits of migration, while addressing its risks and challenges for individuals and communities in countries of origin, transit and destination (UN 2018).

2 See Hillman and Long (2022) for a recent contribution discussing why immigration policies in western democracies often contrast with theoretical predictions of international economics. In particular, they highlight that political parties consider the role of immigrants as future voters when proposing migration policies.

3 See Mazza and Winden (1996) for an early theoretical contribution analyzing the effects of labor migration on income redistribution in a two country model with internationally mobile labor.

4 See Dustmann et al. (2016) for an interesting discussion about the possible reasons for the conflicting evidence on the effects of migration.

5 The difference in endowments between the two groups can be interpreted in various ways reflecting varying economic conditions or differences in productivity. Such differences can be due to educational levels, institutional settings, infrastructure, or other factors.

6 The design of the uc-pgg differs in one crucial aspect from the standard voluntary contribution public good game (vc-pgg). See Appendix D.1 for a detailed discussion and references to related literature.

7 Given the parameters in the experiment, migrating subjects are always better off compared to remaining in group low. In the best-case scenario (contribution equals 100%), a subject earns 15 taler in group low whereas she earns 20 taler in the worst-case scenario (0% contribution) in group high.

8 See “Treatment manipulations” for more information on the migration vote and its differences between treatments.

9 In this respect, our design differs from that of DeAngelo et al. (2020) in which migrants cannot immediately integrate into the labor market and, therefore, cannot contribute to the production of a public good but have to rely on social benefits first.

10 With a changing number of subjects in a group, also the marginal per capita returns (mpcr), i.e., the multiplier divided by the number of subjects in a group changes. We decided to change the multiplier accepting changes in the mpcr but avoiding the need to change other parameters, see Isaac and Walker (1988). See Appendix D.2 for a detailed discussion.

11 Note, that in each experimental session we invited 24 subjects, i.e., three cohorts. Thus, in each session, each multiplier is chosen once as we implemented picking without replacement from the deck of three cards, one showing 1.2, one 1.5 and the third one 1.8.

12 See Kesternich et al. (2018) for a public good experiment with heterogeneous endowments and random and effort-based allocation of endowments.

13 The final payout from the experiment includes the earnings of part 1 (eet) and part 2 (uc-pgg with migration mechanism). The taler earnings in part 2 of the experiment are divided by 20 to get the euro payoff.

14 The results for the decreasing multiplier setting relate to DeAngelo et al. (2020) who report that subjects in their experiment are less willing to accept out-group members if their participation implies a negative impact on the in-group members in a vc-pgg, i.e., benefiting from the public good without being able to contribute to its generation.

15 Decreasing vs. constant: $z = 13.95$, $p =.0000$; decreasing vs. increasing: $z = 20.45$, $p =.0000$; decreasing vs. constant: $z = 8.50$, $p =.0000$.

16 On average, subjects solved 15 sliders with a standard deviation of 6 and a maximum score of 33. That is slightly less than reported in the literature; e.g., average scores of 22 in Gill and Prowse (2012), 17 in Lezzi et al. (2015), and 24 in Araujo et al. (2016). Table C.4 in Appendix C shows an ordinary least squares regression analyzing the score in the real effort task of the et treatments with unconditional voting (model 1).

17 Altruistic-like preferences include kiss-up, inequality averse, maximin or altruistic preferences. Subjects characterized by these preferences care about other people’s payoffs and have positive utility if other agents’ payoffs increase, exceed individual payoffs or have disutility if payoffs vary across agents. Selfish-like preferences include spiteful, kick-down, equality averse, envious or selfish preferences. Agents who are described by these preferences care more about their own payoff than about other people’s well-being. They are either neutral towards other people’s payoffs, have disutility for increasing payoffs of others or positive utility for unequally distributed payoffs across agents. For a detailed description of the different types of distributional preferences we refer to Kerschbamer (2015). We exclude subjects from the analysis that report inconsistent answers in the eet following the convention outlined in Holzmeister and Kerschbamer (2019).

18 Some earlier studies found that individual behavior is affected by entitlement considerations (e.g., Hoffman et al. 1994; Frohlich et al. 2004; Jakiela 2011; Cappelen et al. 2013; Jakiela 2015). However, none of these settings is fully comparable to ours, as we follow a different approach to assess the impact of earned entitlement on subjects’ individual behavior. The cited studies mostly measure distributive preferences in dictator games with earned income from pre-conducted real effort tasks.

19 Consistent with what we have discussed previously for Fig. 1, Table C.4 (see Appendix C, models 1 to 3) shows a significant treatment effect for $\textsc {cer\_rand}$ compared to $\textsc {unc\_rand}$ only for the decreasing multiplier case. Even this effect is not robust once we include further control variables to the logistic regression, like in Table 3, and occurs only for the decreasing multiplier case.

20 See Ledyard (1995) and Chaudhuri (2011) for detailed literature reviews on the evolution of public good games. Examples with much lower contribution rates include, e.g., Marwell and Ames (1979); Andreoni (1995); and Bochet et al. (2006). Figures C.1 to C.4 in Appendix C show median contribution levels for each cohort, period, and treatment.

21 Note that all significance tests are done with the Wilcoxon rank-sum test except when stated otherwise.

22 Similarly, as seen in Table C.3 (model 1, Appendix C), we do not find significant effects of proposed contribution levels between groups, change, and no-change cohorts and different levels of mpcr, respectively.

23 We exclude subjects from the analysis that report inconsistent answers in the eet following the convention outlined in Holzmeister and Kerschbamer (2019).

24 As seen in Table C.3 (Appendix C), the reported effects are stable even when controlling for other variables, e.g., groups, change and no-change cohorts, and different levels of mpcr.
