# Supplementary material for: Acceptance or rejection of welfare migration—an experimental investigation
Source: SN Bus Econ. 2022 Oct 24;2(11):179. doi: 10.1007/s43546-022-00356-6 (PMC9592661; doi:10.1007/s43546-022-00356-6)
Supplement: Supplementary file 1 — Supplementary file1 (PDF 602 KB) [file 43546_2022_356_MOESM1_ESM.pdf]

Appendix

A Instructions for the experiment

Section A.1 and A.2 provide translated instructions for the first part and the main part of the experiment, respectively. In the first part, the EET is described. In sections A.2.1 and A.2.2 instructions for the treatment manipulations CER and UNC are provided. Original instructions in German language are available upon request. Variations of instructions of treatment manipulations ET and RAND are displayed in [square brackets] including an indication regarding the corresponding treatment. In the experiment, we varied the group labelling for half of the cohorts such that group HIGH is labelled group A for half of the cohorts and group B for the other half and vice versa.

A.1 First part of the experiment: EET

Dear participant!
We would like to welcome you to this experiment. We kindly ask you to henceforth not talk to any person except the experimenter. If you have any questions regarding the instructions and the procedure of the experiment, please raise your hand and your question will be privately answered by the experimenter.

Additionally, please note the following:

- All statements written in this instruction are true.
- Each participant receives the same instructions and information about the experiment.
- Your decisions are anonymous.
- You are not allowed to use your mobile phone or calculator. Furthermore, you are only allowed to use the functions of the computer, which are necessary for conducting the experiment. If you do not follow these rules of conduct, you will NOT get any payout from the experiment.

Experimental procedure
This session consists of two independent experiments. You can earn money in both of the following experiments. Each instruction is handed out before the respective experiment starts.

- Experiment 1
  - Instruction
  - Experiment
- Experiment 2
  - Instruction
  - Experiment

– Questionnaire

- Payment (sum of payouts from experiment 1 and experiment 2)

Instruction of Experiment 1
Experiment 1 consists of 10 decisions. During these 10 decision situations you are randomly and anonymously matched with one other participant in this room. The person you are matched with will be called „Player B" during the experiment. IN EACH OF THE 10 DECISION SITUATIONS you (and the other player) have to choose between option LEFT and option RIGHT. Each decision has consequences for your own and player B's payout.

Example: You are asked whether you prefer option LEFT where you receive a payout of €3.00 and player B receives €7.00 or option RIGHT where each of you, i.e., you and player B, each receive €4.00. You are then asked to make a decision. The decision situation is displayed as follows:

Option „LEFT"				Option „RIGHT"	
Your Payoff	*Payoff Player B*	*Make your decision here*		*Your Payoff*	*Payoff Player B*
3,00 €	7,00 €	○	○	4,00 €	4,00 €

In this experiment you will make 10 such decisions. Your total payout from this experiment is determined the following: At the end of experiment 1 one decision situation is chosen for payout by a random mechanism. Additionally, it is determined whether your payout results from your own decision (as an active person) or the decision of another player (as player B). For example, if based on the random mechanism it is decided that your payout is determined by your own decision (as active person) and the above decision situation is chosen, you and your player B would receive €4.00 each in case RIGHT was chosen. If LEFT was chosen, you would receive €3.00 and player B would receive €7.00.

A.2 Main experiment

A.2.1 Treatment manipulation CER

Introduction to Experiment 2
In this experiment, eight participants form a cohort to then interact over eight periods. At the beginning of the experiment, you are assigned to one of two groups (A and B). Group A *(B)* consists of five participants and group B *(A)* consists of three participants. Each participant receives a constant endowment in taler in each period. However, the amount of taler participants receive differs between groups A and B. Participants in group A *(B)* receive 20 taler as period endowment and participants in group B *(A)* receive 10 taler as period endowment. In each period, each subject within a group makes a proposal on the percentage of the periodic endowment she and everybody else in her group should contribute to a public pool. All proposals within a group are collected and the median of the proposals becomes the contribution level that each subject

pays into the public pool. The sum of all contributions is then multiplied with a factor of 1.5 and is then paid to each participant in equal parts.

Furthermore, there is the possibility for participants of group B *(A)* to change to group A *(B)* in period five. In the following, you receive detailed information on the group assignment mechanism, the different tasks and the possibility of a group change.

Group assignment

[ET: The group assignment is conducted according to the outcome of a real effort task, where each participant sees several sliders. At the beginning of the task each slider is placed on a value of 0 but it can be moved to any value between 0 and 100 by using the computer mouse (see Figure A.1). The aim of the task is to move as many sliders to the value of 50 as possible. The score of each participant in the task equals the number of sliders positioned at 50.

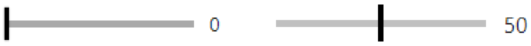

Figure A.1: Initial slider position (0) and preferred slider position (50).

The slider can be moved by clicking on the left button of the computer mouse. As soon as the button is clicked, the position of the slider is displayed. The slider is solved if it has a position of 50. Otherwise, the slider can be rearranged by clicking again on the left button of the computer mouse to move it to a value of 50. Before the actual slider task is played, each participant has the opportunity to familiarize with the mechanism of the slider task and additionally participants are asked to estimate the probability of being in group A *(B)*. After that, the actual slider task is conducted: In total, 48 sliders are displayed and each participant has 2 minutes to position as many sliders as possible on a value of 50. Each participant is then assigned either to group A or group B. The five participants with the highest number of correctly positioned sliders are assigned to group A *(B)*, the other three participants are assigned to group B *(A)*. Any ties due to equal numbers of correctly positioned sliders are resolved by a random mechanism.]
[RAND: The group assignment at the beginning of the experiment is conducted randomly with five people assigned to group A *(B)* and three assigned to group B *(A)*. Before the group assignment participants are asked to estimate the probability of being in group A *(B)*.]

Preferences regarding the group change

After the group assignment, each participant of group A *(B)* anonymously states her individual preferences regarding a potential group change of one participant of group B *(A)* to your group A *(B)*. The potential group change takes place in period 5. However, when a group change occurs, the factor with which the contributions to the public pool are multiplied can change either to 1.2 (scenario 1), stay at 1.5 (scenario 2) or change to 1.8 (scenario 3). Each participant of group A *(B)* states her preferences whether to approve a group change or not for each single scenario. A group change is approved if the majority in group A *(B)* votes in favor of a group change. If there is no group change due to the stated preferences, the factor to multiply the contributions to the public pool stays at 1.5. In the meantime, participants of group B *(A)* are asked to estimate the percentage probability for each scenario that participants of group A *(B)*

vote in favor of a group change.

Contribution to the public pool

Each group, i.e., group A and B, has a common public pool, and participants can decide how much of their periodic endowment they want to contribute to the pool. The contribution is determined by a vote, which is conducted at the beginning of each round. The defined contribution is binding for each participant. Each participant proposes a contribution level, which can be stated between 0% and 100% (in steps of 10 percentage points) of the periodic endowment. The mandatory contribution for each participant is determined by the median of all proposed contributions stated by each participant, i.e., the mandatory contribution is the (ascending sorted) contribution which makes up the middle value. If the amount of participants within a group is even due to a group change, then the median is defined by the mean of the two middle values. For clarification, Table tab:Numerical examples of how to calculate the median. provides some numerical examples of how the median is calculated.

Contributions (sorted)	1	2	3	4	5	6	Median
Example 1	80%	80%	80%	80%	80%	-	80%
Example 2	20%	30%	70%	80%	80%	80%	75%
Example 3	30%	40%	50%	-	-	-	40%
Example 4	50%	80%	-	-	-	-	65%

Table A.1: Numerical examples of how to calculate the median.

Computation of the period earnings

After the vote regarding the proposed contributions, each participant has to contribute the median of those stated contributions to a public pool. The sum of these contributions is multiplied by a factor of 1.5 and is paid out to each participant within a group in equal parts. The following equation thus shows the computation of the period earnings.

$$\text{Period Earnings in taler} = \text{Period Endowment} - \text{Contribution to the Public Pool} + \frac{(\text{Sum Contributions} \cdot 1.5)}{(\text{Amount Participants})} \quad (\text{A.1})$$

Table A.2 provides some examples regarding the computation of the period earnings for different contribution levels to the public pool. The period earnings of the previous periods are put into a separate account and are then used to calculate the final payout from the experiment (more information is provided at the end of the instructions).

	Group A *(B)*			Group B *(A)*		
Contribution	20%	50%	80%	20%	50%	80%
Period Endowment	20.0	20.0	20.0	10.0	10.0	10.0
Contribution to the Public Pool (taler)	4.0	10.0	16.0	2.0	5.0	8.0
Sum of Contributions	20.0	50.0	80.0	6.0	15.0	24.0
Sum of Contributions ·1.5	30.0	75.0	120.0	9.0	22.5	36.0
Number of Participants	5	5	5	3	3	3
Payout from the Public Pool	6.0	15.0	24.0	3.0	7.5	12.0
Period Earnings	22.0	25.0	28.0	11.0	12.5	14.0

Table A.2: Numerical examples for the computation of the period earnings for group A and group B.

Group change in period 5

At the beginning of period 5 it is determined, which scenario applies for your cohort. For this purpose, we have three cards where either the factor 1.2, 1.5 or 1.8 is written. One participant of your cohort is asked to blindly pick one card. The value, which is written on the card, then determines the new factor in case of a group change. The new factor is displayed on the screen. After that, it is determined whether according to the vote in period 1 participants of group A *(B)* approved a group change for the drawn scenario or not. If an ordinary majority (3 out of 5 participants) voted in favor of a group change for the drawn scenario, one randomly chosen participant of group B *(A)* changes to group A *(B)* and the new factor is applied. If there is no majority who approved a group change, then the group size and the factor do not change. Please note, that the factor for group B *(A)* never changes.

Computation of your final payoff

Your final payout from this experiment is determined by the sum of the period earnings which are put into a separate account after each period. The sum of all period earnings is divided by 20 which then determines your euro payout (rounded to 50 eurocents).

$$\text{Payout in euro} = \frac{\text{(Sum of all Period Earnings)}}{20} \tag{A.2}$$

Important Information

- The experiment consists of eight periods.
- [ET: The higher your scores in the slider task, the more likely it is that you are assigned to group A *(B)*.]
- [RAND: Groups are randomly assigned.]

- Each participant in group A *(B)* gets a period endowment of 20 taler and each participant of group B (A) receives a period endowment of 10 taler.
- There is a possibility that one participant of group B *(A)* changes to group A *(B)* in period 5.
- The factor to multiply the contributions in the public pool can change in case of a group change.

A.2.2 Treatment manipulation UNC

Introduction to Experiment 2
In this experiment, eight participants form a cohort to then interact over eight periods. At the beginning of the experiment, you are assigned to one of two groups (A and B). Group A *(B)* consists of five participants and group B *(A)* consists of three participants. Each participant receives a constant endowment in taler in each period. However, the amount of taler participants receive differs between groups A and B. Participants in group A *(B)* receive 20 taler as period endowment and participants in group B *(A)* receive 10 taler as period endowment. In each period, each subject within a group makes a proposal on the percentage of the periodic endowment she and everybody else in her group should contribute to a public pool. All proposals within a group are collected and the median of the proposals becomes the contribution level that each subject pays into the public pool. The sum of all contributions is then multiplied with a factor of 1.5 and is then paid to each participant in eual parts. Additionally, there is the possibility for participants of group B *(A)* to change to group A *(B)* in period five. In the following, you receive detailed information on the group assignment mechanism, the different tasks and the possibility of a group change.

Preferences regarding the group change
At the beginning of period 5, one participant of group B *(A)* is potentially allowed to change to group A *(B)*. However, in case of a group change, the factor to multiply the contributions to the public pool can change either to 1.2 (scenario 1), stay at 1.5 (scenario 2) or change to 1.8 (scenario 3). To determine whether a group change takes place or not each participant states her preferences whether to approve a group change or not for each single scenario. If there is no group change due to the stated preferences, the factor to multiply the contributions to the public pool stays at 1.5.

Group assignment
[ET: The group assignment is conducted according to the outcome of a real effort task, where each participant sees several sliders. At the beginning of the task each slider is placed on a value of 0 but it can be moved to any value between 0 and 100 by using the computer mouse (see Figure A.2). The aim of the task is to move as many sliders to the value of 50 as possible. The score of each participant in the task equals the number of sliders positioned at 50.

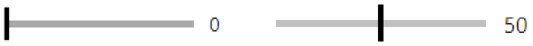

Figure A.2: Initial slider position (0) and preferred slider position (50).

The slider can be moved by clicking on the left button of the computer mouse. As soon as the button is clicked, the position of the slider is displayed. The slider is solved if it has a position of 50. Otherwise, the slider can be rearranged by clicking again on the left button of the computer mouse to move it to a value of 50. Before the actual slider task is played, each participant has the opportunity to familiarize with the mechanism of the slider task and additionally participants are asked to estimate the probability of being in group A *(B)*. After that, the actual slider task is conducted: In total, 48 sliders are displayed and each participant has 2 minutes to position as many sliders as possible on a value of 50. Each participant is then assigned either to group A or group B. The five participants with the highest number of correctly positioned sliders are assigned to group A *(B)*, the other three participants are assigned to group B *(A)*. Any ties due to equal numbers of correctly positioned sliders are resolved by a random mechanism.]
[RAND: The group assignment at the beginning of the experiment is conducted randomly with five people assigned to group A *(B)* and three assigned to group B *(A)*. Before the group assignment participants are asked to estimate the probability of being in group A *(B)*.]

Contribution to the public pool
Each group, i.e., group A and B, has a common public pool, and participants can decide how much of their periodic endowment they want to contribute to the pool. The contribution is determined by a vote, which is conducted at the beginning of each round. The defined contribution is binding for each participant. Each participant proposes a contribution level, which can be stated between 0% and 100% (in steps of 10 percentage points) of the periodic endowment. The mandatory contribution for each participant is determined by the median of all proposed contributions stated by each participant, i.e., the mandatory contribution is the (ascending sorted) contribution which makes up the middle value. If the amount of participants within a group is even due to a group change, then the median is defined by the mean of the two middle values. For clarification, Table A.3 provides some numerical examples of how the median is calculated.

Contributions (sorted)	1	2	3	4	5	6	Median
Example 1	80%	80%	80%	80%	80%	-	80%
Example 2	20%	30%	70%	80%	80%	80%	75%
Example 3	30%	40%	50%	-	-	-	40%
Example 4	50%	80%	-	-	-	-	65%

Table A.3: Numerical examples of how to calculate the median.

Computation of the period earnings
After the vote regarding the preferred contributions, each participant has to contribute the

median of those proposed contributions to a public pool. The sum of these contributions is multiplied by a factor of 1.5 and is paid out to each participant within a group in equal parts. The following equation thus shows the computation of the period earnings.

$$\text{Period Earnings in taler} = \text{Period Endowment} - \text{Contribution to the Public Pool} + \frac{(\text{Sum Contributions} \cdot 1.5)}{(\text{Amount Participants})} \quad \text{(A.3)}$$

Table A.4 provides some examples regarding the computation of the period earnings for different contributions to the public pool. The period earnings of the previous periods are put into a separate account and are then used to calculate the final payout from the experiment (more information is provided at the end of the instructions).

	Group A *(B)*			Group B *(A)*		
Contribution	20%	50%	80%	20%	50%	80%
Period Endowment	20.0	20.0	20.0	10.0	10.0	10.0
Contribution to the Public Pool (taler)	4.0	10.0	16.0	2.0	5.0	8.0
Sum of Contributions	20.0	50.0	80.0	6.0	15.0	24.0
Sum of Contributions ·1.5	30.0	75.0	120.0	9.0	22.5	36.0
Number of Participants	5	5	5	3	3	3
Payout from the Public Pool	6.0	15.0	24.0	3.0	7.5	12.0
Period Earnings	22.0	25.0	28.0	11.0	12.5	14.0

Table A.4: Numerical examples for the computation of the period earnings for group A and group B.

Group change in period 5

At the beginning of period 5 it is determined, which scenario applies for your cohort. For this purpose, we have three cards where either the factor 1.2, 1.5 or 1.8 is written. One participant of your cohort is asked to blindly pick one card. The value, which is written on the card, then determines the new factor in case of a group change. The new factor is displayed on the screen. After that, it is determined whether according to the vote in period 1 participants of group A *(B)* approved a group change for the drawn scenario or not. If an ordinary majority (3 out of 5 participants) voted in favor of a group change for the drawn scenario, one randomly chosen participant of group B *(A)* changes to group A *(B)* and the new factor is applied. If there is no ordinary majority who approved a group change, then the group size and the factor do not change. Please note, that the factor for group B *(A)* never changes.

Computation of your final payoff

Your final payout from this experiment is determined by the sum of the period earnings, which are put into a separate account after each period. The sum of all period earnings is divided by 20 which then determines your euro payout (rounded to 50 eurocents).

$$\text{Payout in euro} = \frac{\text{(Sum of all Period Earnings)}}{20} \tag{A.4}$$

Important Information

- The experiment consists of eight periods.
- [ET: The higher your score in the slider task, the more likely it is that you are assigned to group A *(B)*.]
- [RAND: Groups are randomly assigned.]
- Each participant in group A *(B)* gets a period endowment of 20 taler and each participant of group B *(A)* receives a period endowment of 10 taler.
- There is a possibility that one participant of group B *(A)* changes to group A *(B)* in period 5.
- The factor to multiply the contributions in the public pool can change in case of a group change.

B Screenshots from the software

Option 'Left'				Option 'Right'	
Your Payoff	Player B's Payoff			Your Payoff	Player B's Payoff
€2.00	€1.00	○	○	€4.00	€4.00
€3.00	€1.00	○	○	€4.00	€4.00
€4.00	€1.00	○	○	€4.00	€4.00
€5.00	€1.00	○	○	€4.00	€4.00
€6.00	€1.00	○	○	€4.00	€4.00

Next

Figure B.1: First decision screen in the Equality Equivalence Test

Option 'Left'				Option 'Right'	
Your Payoff	Player B's Payoff			Your Payoff	Player B's Payoff
€2.00	€7.00	○	○	€4.00	€4.00
€3.00	€7.00	○	○	€4.00	€4.00
€4.00	€7.00	○	○	€4.00	€4.00
€5.00	€7.00	○	○	€4.00	€4.00
€6.00	€7.00	○	○	€4.00	€4.00

Next

Figure B.2: Second decision screen in the Equality Equivalence Test

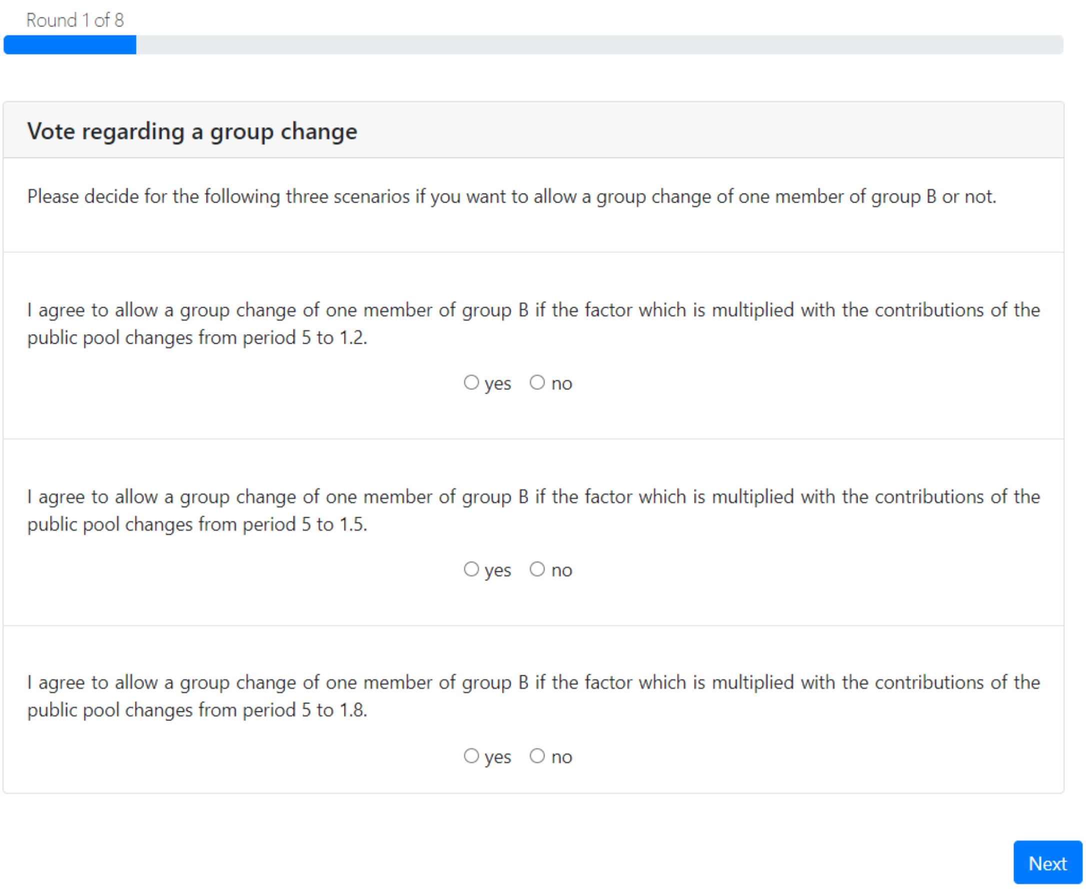

Figure B.3: Decision screen for subjects in group HIGH regarding the vote of a group change in each scenario in treatments CER_RAND and CER_ET.

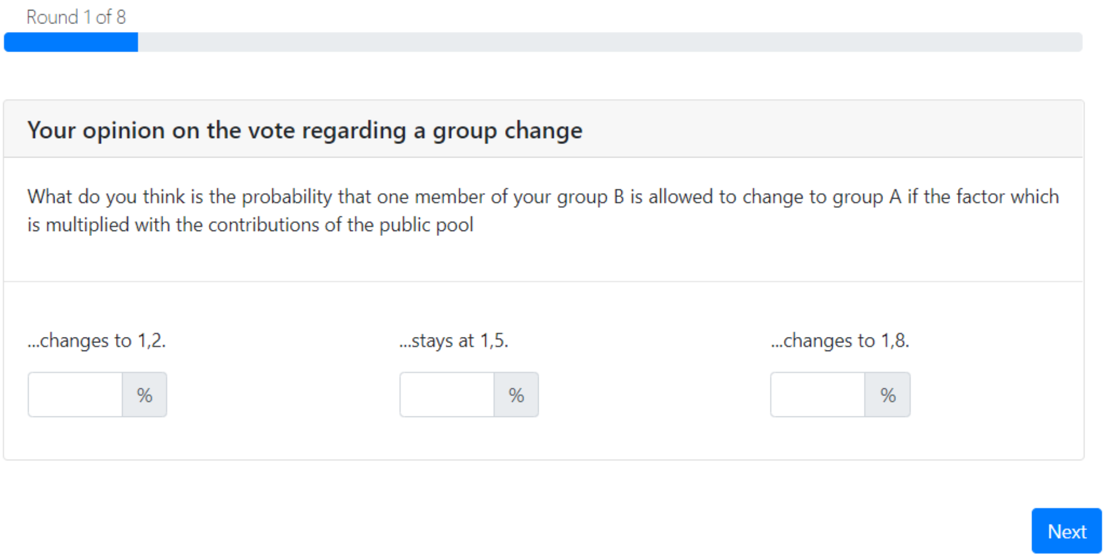

Figure B.4: Decision screen of subjects in group LOW regarding the probability of a group change in each scenario in treatments CER_RAND and CER_ET.

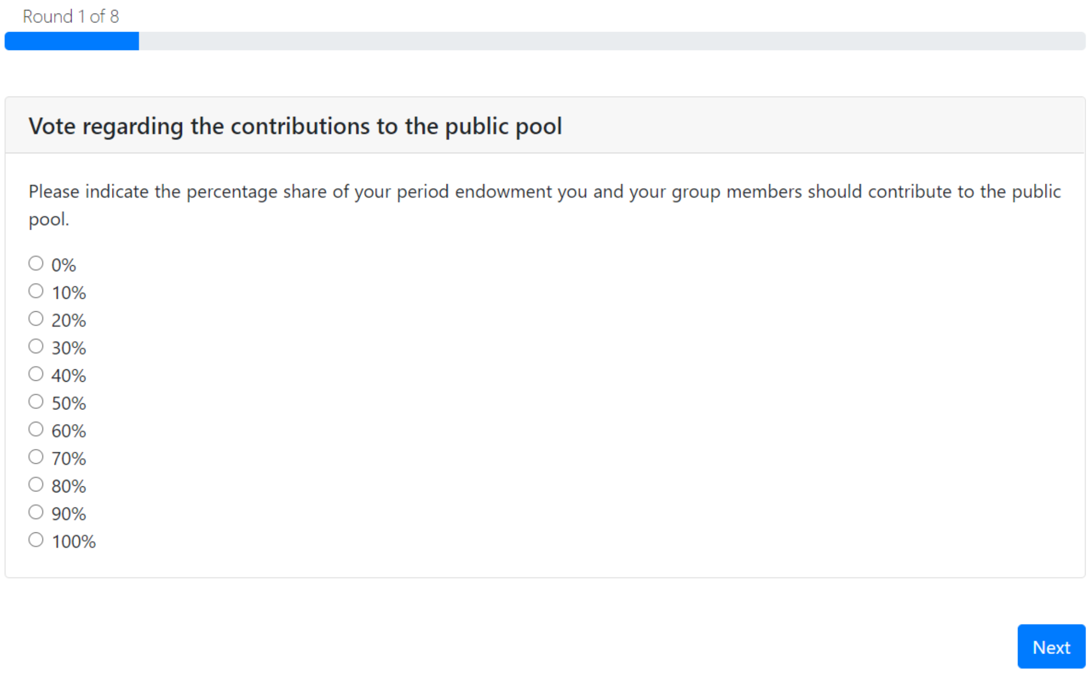

Figure B.5: Decision screen regarding subjects' preferred contributions.

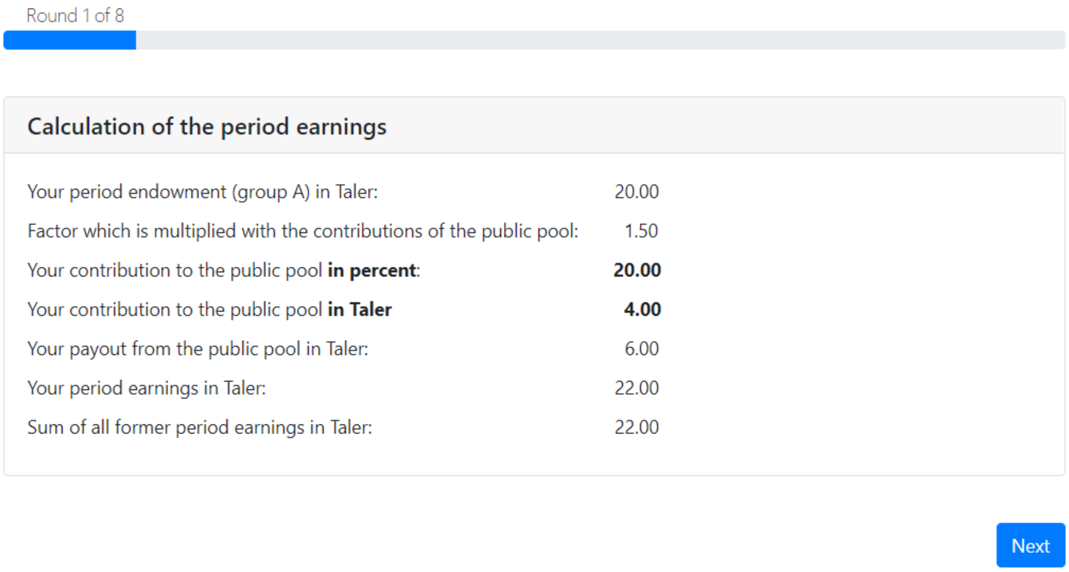

Figure B.6: History screen.

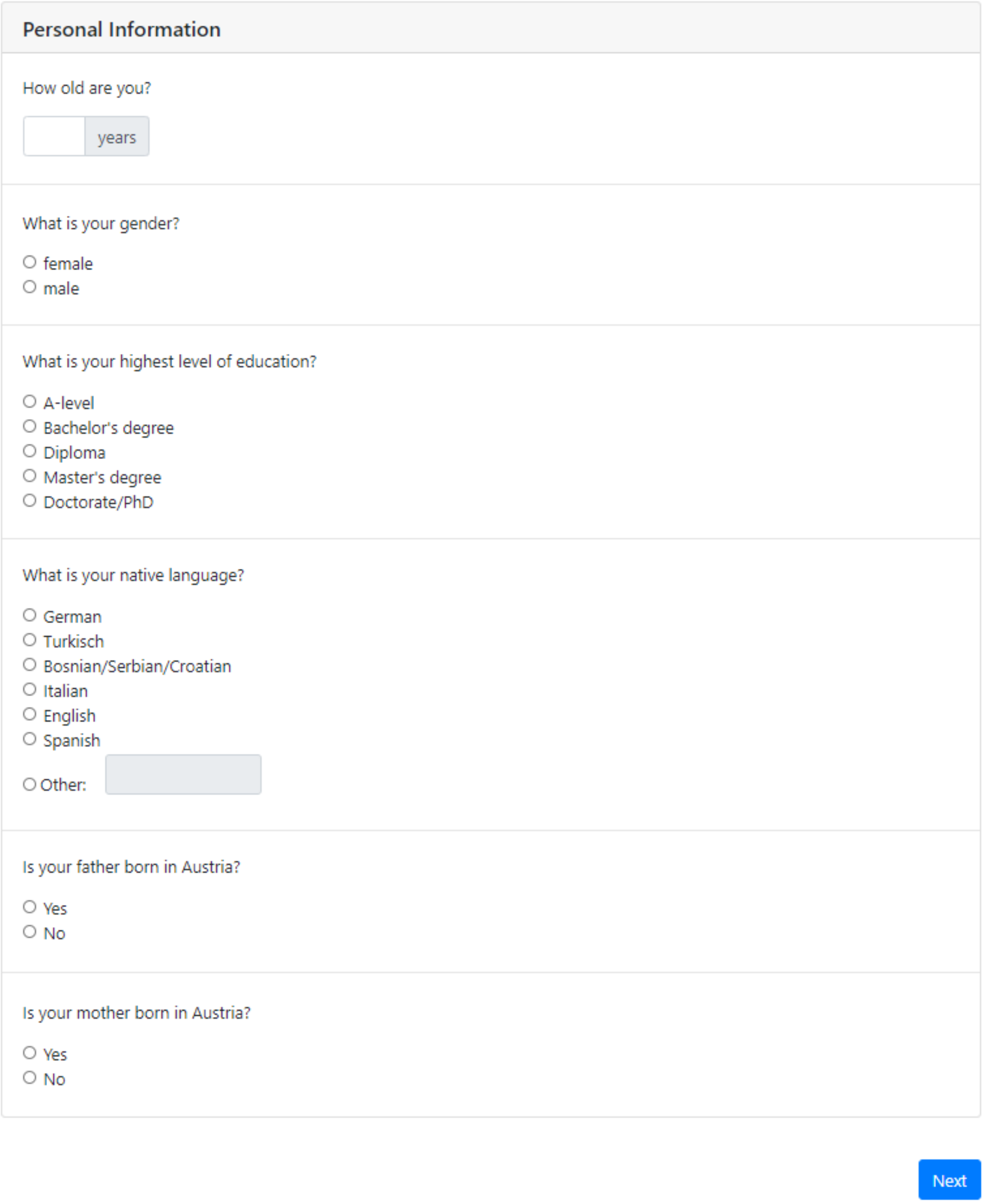

Personal Information

How old are you?

years

What is your gender?

- female
- male

What is your highest level of education?

- A-level
- Bachelor's degree
- Diploma
- Master's degree
- Doctorate/PhD

What is your native language?

- German
- Turkisch
- Bosnian/Serbian/Croatian
- Italian
- English
- Spanish
- Other:

Is your father born in Austria?

- Yes
- No

Is your mother born in Austria?

- Yes
- No

Next

Figure B.7: Demographics screen I.

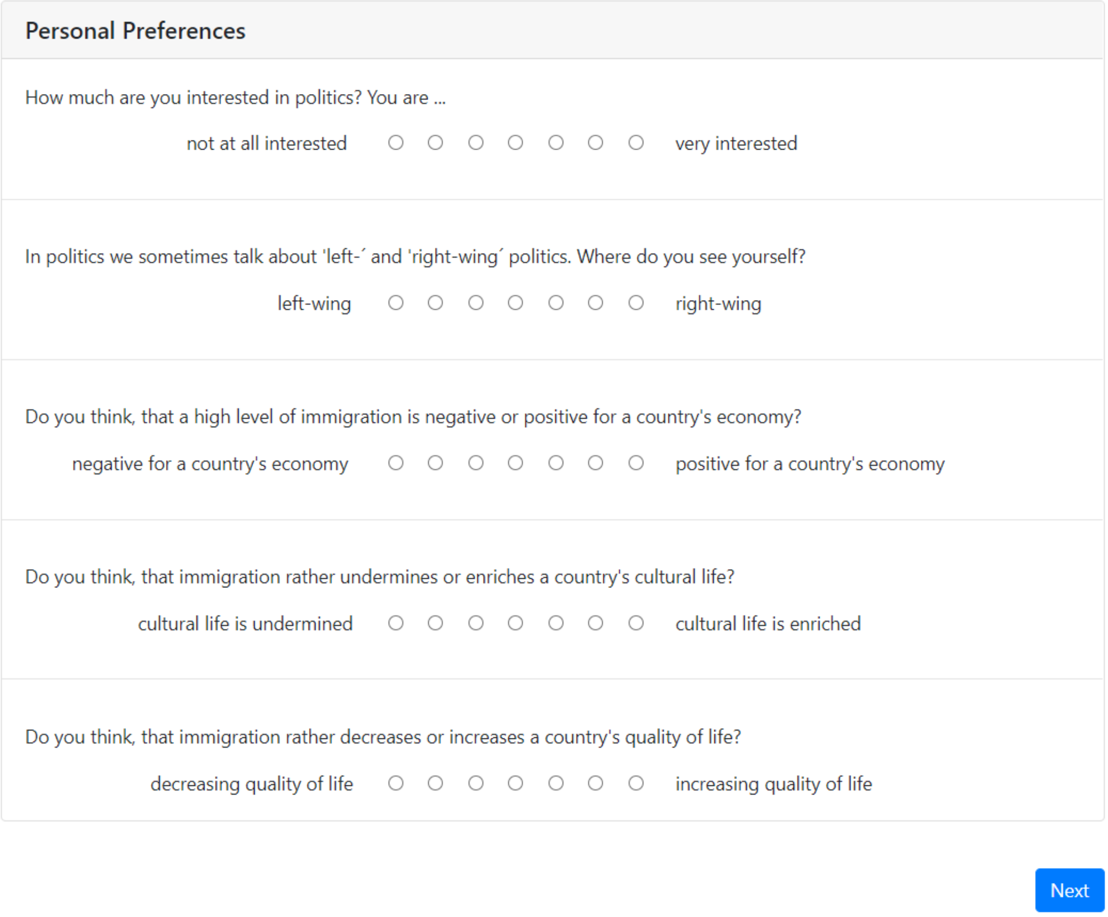

Figure B.8: Demographics screen II.

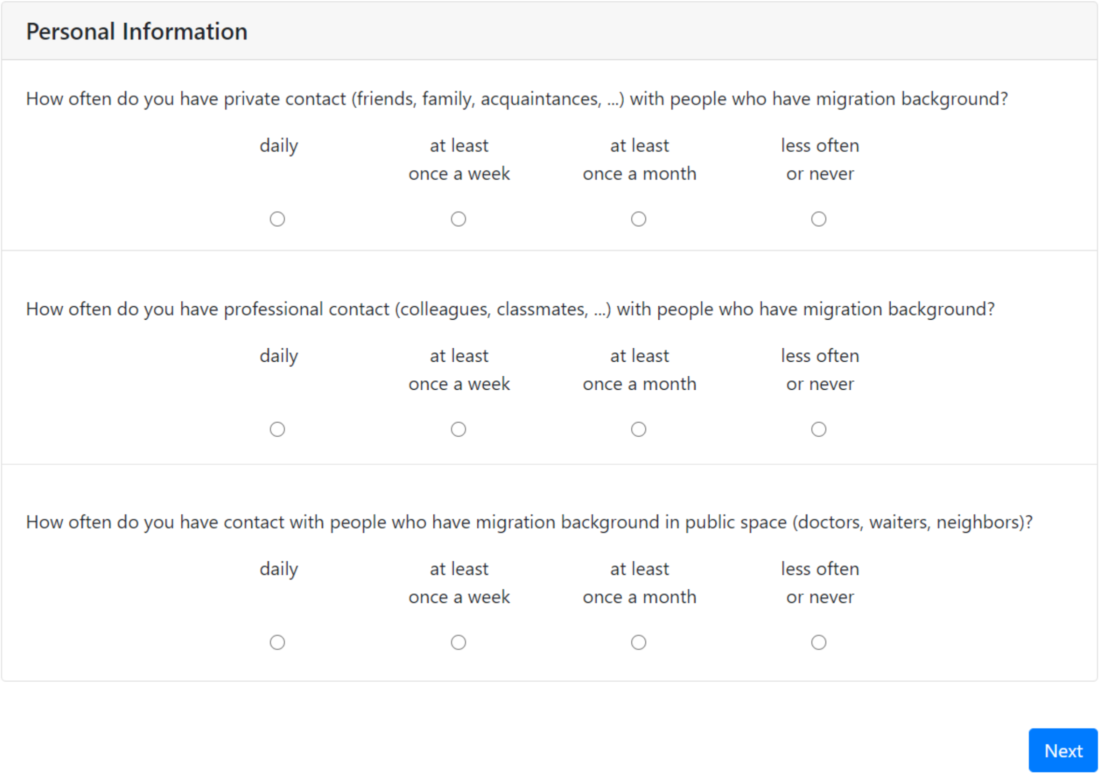

Personal Information

How often do you have private contact (friends, family, acquaintances, ...) with people who have migration background?

daily	at least once a week	at least once a month	less often or never
○	○	○	○

How often do you have professional contact (colleagues, classmates, ...) with people who have migration background?

daily	at least once a week	at least once a month	less often or never
○	○	○	○

How often do you have contact with people who have migration background in public space (doctors, waiters, neighbors)?

daily	at least once a week	at least once a month	less often or never
○	○	○	○

Next

Figure B.9: Demographics screen III.

C Additional figures and tables

Table C.1: **Overview of sample sizes and descriptive statistics.** The upper panel of the table provides an overview about the number of cohorts (# cohorts), the group (HIGH, LOW) and cohort sizes, the total number of participating subjects (# subjects), and the number of voting subjects (# voting subjects) conditional on voting mechanisms and treatment conditions. The lower panel of the table depicts the share of female subjects (female %), the subjects' average age, and the average payoff (payoff €).

	Conditional voting				Unconditional voting	
	CER_RAND	CER_ET	UNC_RAND	UNC_ET	CER_RAND	CER_ET
# cohorts	12	12	12	12	12	12
Group size HIGH	5	5	5	5	5	5
Group size LOW	3	3	3	3	3	3
Cohort size	8	8	8	8	8	8
# subjects	96	96	96	96	96	96
# voting subjects	60	60	96	96	60	60
Female (%)	52.1%	53.1%	53.1%	60.4%	56.3%	50.0%
Age (years)	23.69	22.49	23.19	22.96	22.10	22.14
Payoff (€)	18.65	18.73	18.69	19.30	18.52	18.42

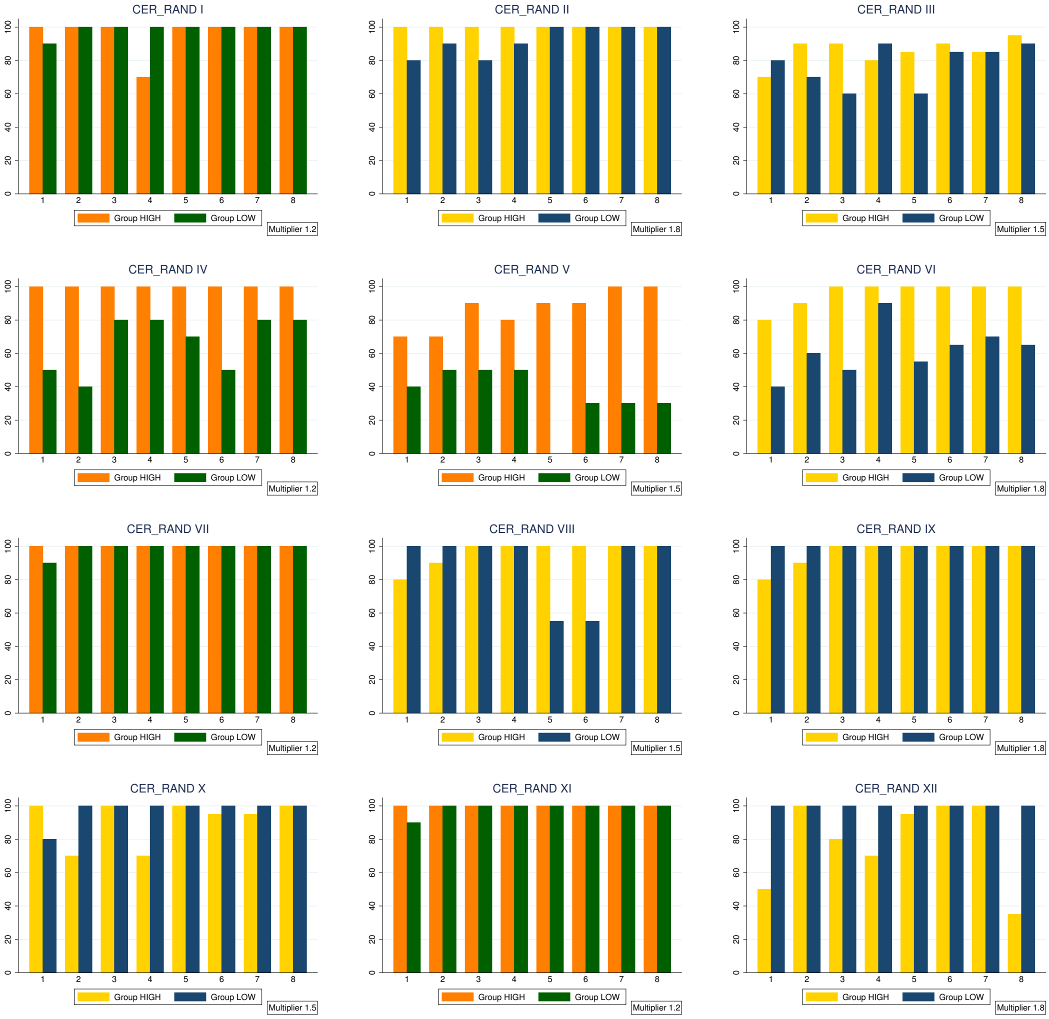

Figure C.1: **Median contribution levels in treatment CER_RAND.** The figure shows median contribution levels for each cohort in each period in treatment CER_RAND. The stated multiplier represents the new multiplier in case of a group change. Bar charts are coloured in yellow and orange for group A and blue and green for group B, respectively. Graphs with yellow and blue bars represent cohorts with a group change in period 5 and graphs with orange and green bars show cohorts without a group change in period 5.

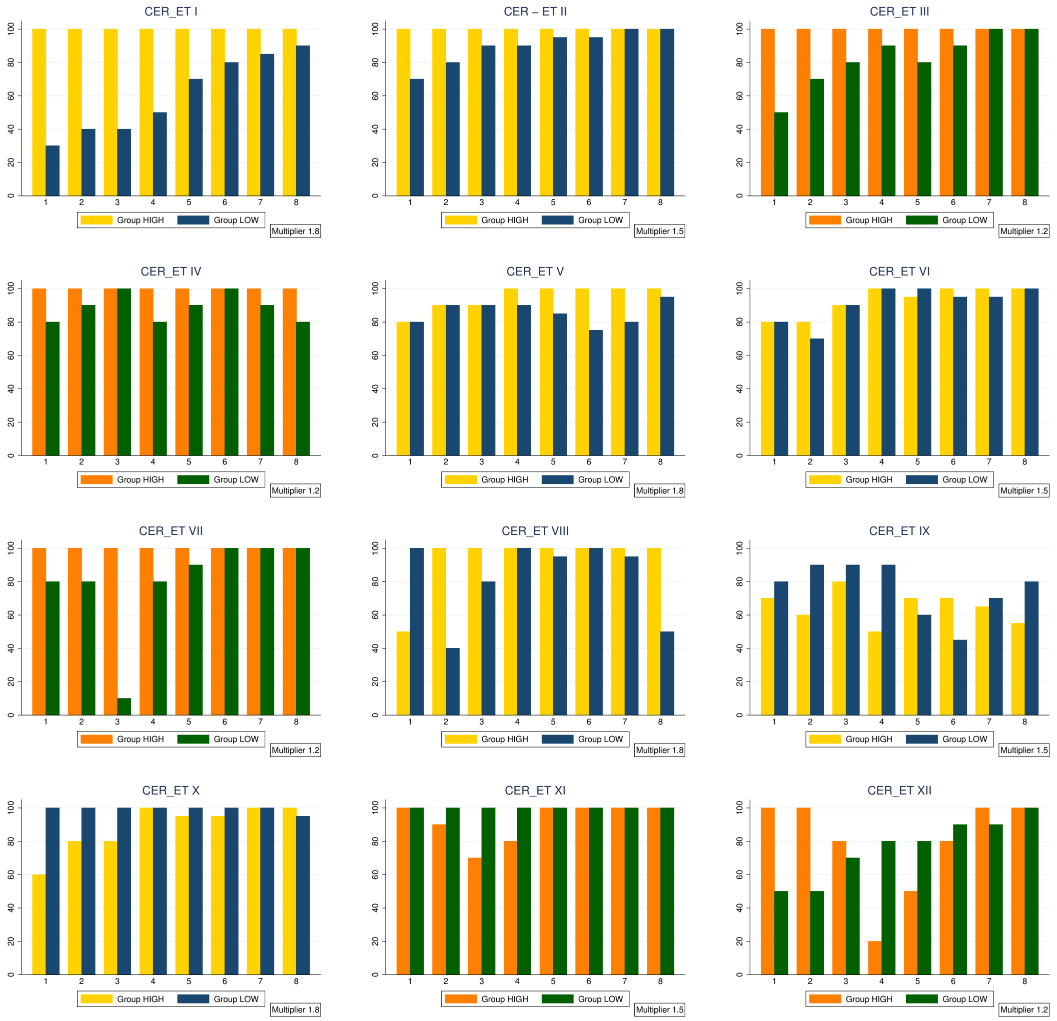

Figure C.2: **Median contribution levels in treatment CER_ET.** The figure shows median contribution levels for each cohort in each period in treatment CER_ET. The stated multiplier represents the new multiplier in case of a group change. Bar charts are coloured in yellow and orange for group A and blue and green for group B, respectively. Graphs with yellow and blue bars represent cohorts with a group change in period 5 and graphs with orange and green bars show cohorts without a group change in period 5.

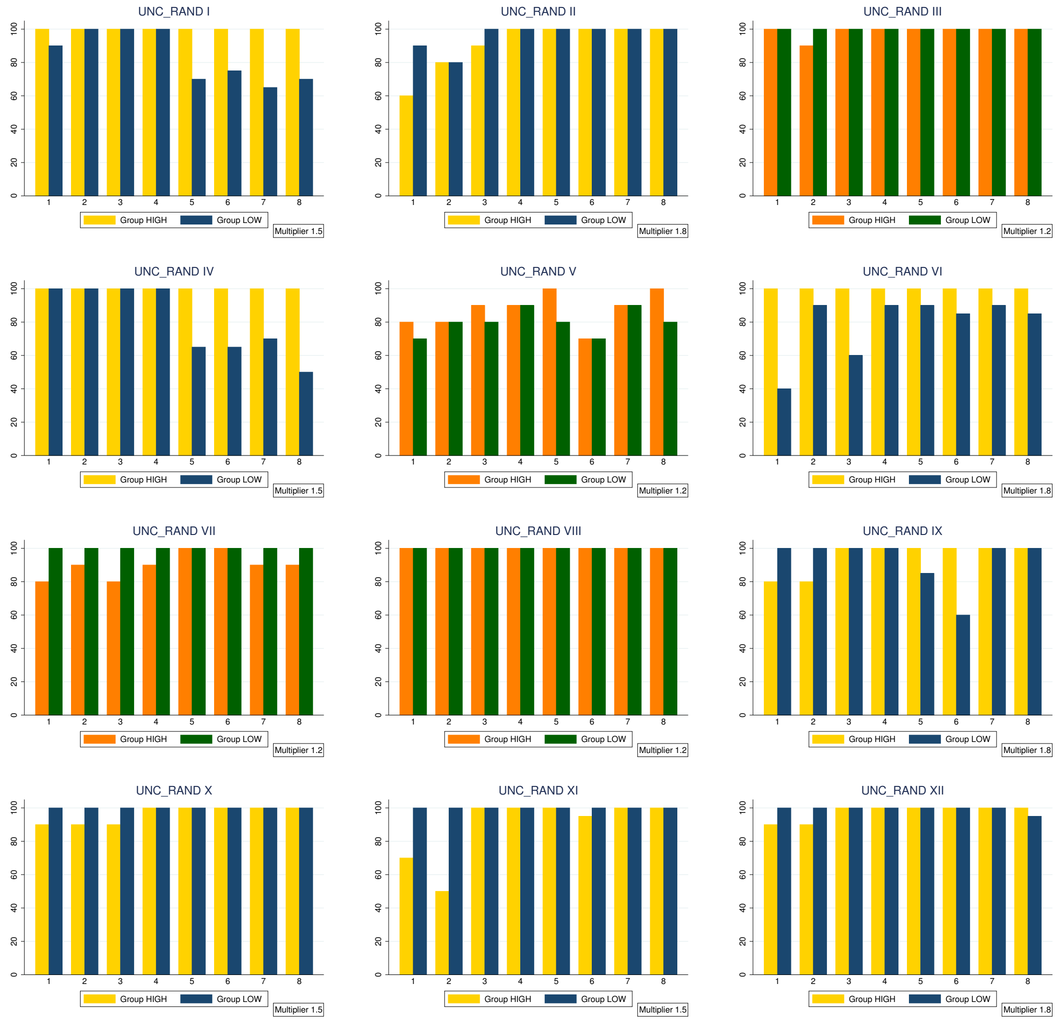

Figure C.3: **Median contribution levels in treatment UNC_RAND.** The figure shows median contribution levels for each cohort in each period in treatment UNC_RAND. The stated multiplier represents the new multiplier in case of a group change. Bar charts are coloured in yellow and orange for group A and blue and green for group B, respectively. Graphs with yellow and blue bars represent cohorts with a group change in period 5 and graphs with orange and green bars show cohorts without a group change in period 5.

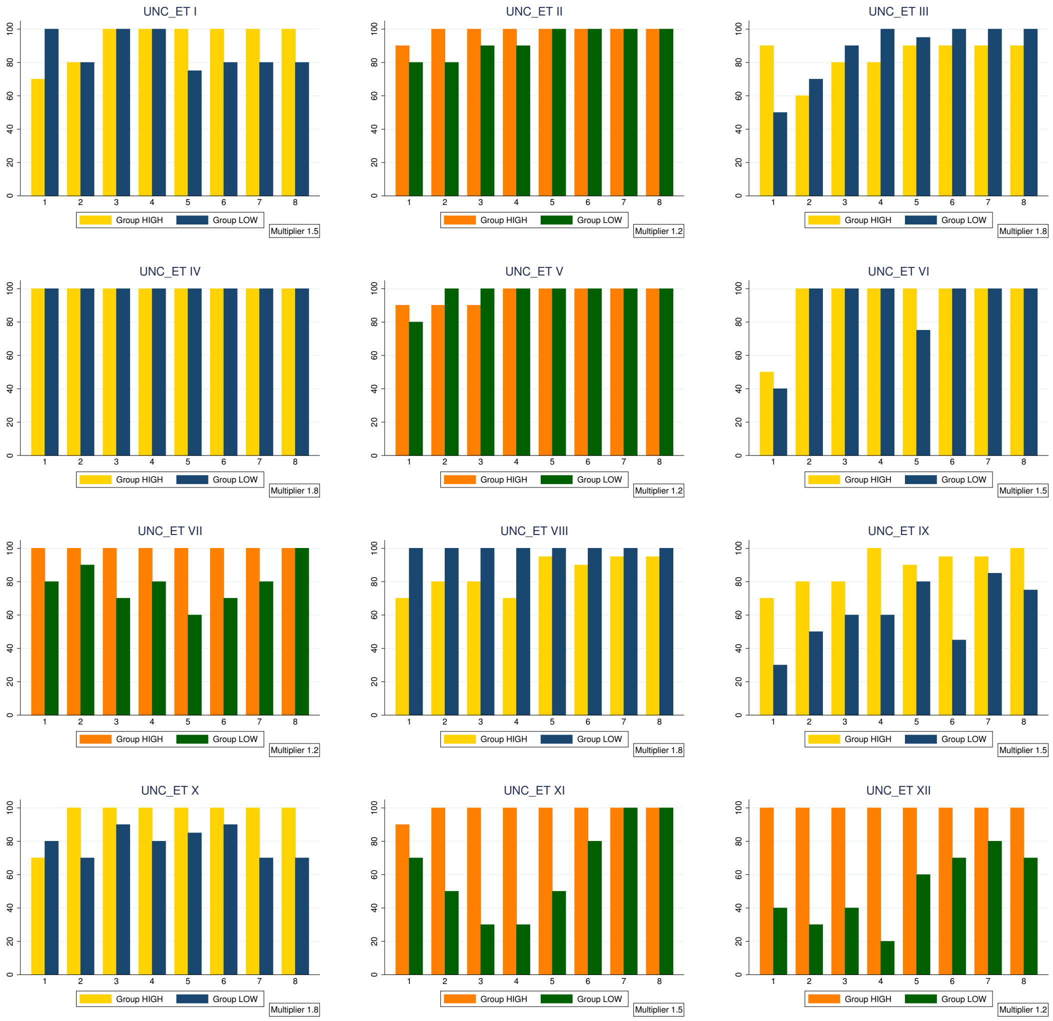

Figure C.4: **Median contribution levels in treatment UNC_ET.** The figure shows median contribution levels for each cohort in each period in treatment UNC_ET. The stated multiplier represents the new multiplier in case of a group change. Bar charts are coloured in yellow and orange for group A and blue and green for group B, respectively. Graphs with yellow and blue bars represent cohorts with a group change in period 5 and graphs with orange and green bars show cohorts without a group change in period 5.

Table C.2: **Logistic regressions explaining subjects' voting behavior in favor or against a group change.** The table shows estimates from logistic regressions with VOTE_DEC (model 1 and 4), VOTE_CONST (model 2 and 5), and VOTE_INC (model 3 and 6) as dependent variables. All three variables are dummy variables which take on a value of 0 if subjects vote against a group change and 1 if subjects vote in favor of a group change. DEC, CONST and INC represent the conditions of a decreasing (1.2), constant (1.5) and increasing (1.8) multiplier. ET and CER are dummies for the treatment manipulations. ET takes on a value of 1 if subjects were assigned to groups by conducting the slider task or 0 if they were randomly assigned. CER is 1 if the vote regarding the group change is conducted after the group assignment and 0 if it takes place before the group assignment. ET X CER is the interaction term for ET and CER and captures the marginal effect. The independent variable FEMALE is a dummy variable and takes on values of 0 for male subjects and 1 for female subjects. POL_PREFERENCE is an ordinal variable from 0 to 6 where 0 means a subject self-identified as rather left–wing, while 6 means a subject self-identified as rather right–wing. ALTRUISTIC-LIKE is a dummy variable taking on values of 0 if subjects' distributional preferences are categorized either as spiteful, kick-down, equality averse, envious or selfish and 1 if their distributional preferences are either kiss-up, inequality averse, maximin or altruistic, respectively. Clustered standard errors on cohort level are in parentheses. *, **, and *** represent the 5%, 1%, and 0.1% significance level, respectively.

	VOTE_DEC	VOTE_CONST	VOTE_INC	VOTE_DEC	VOTE_CONST	VOTE_INC
	(1)	(2)	(3)	(4)	(5)	(6)
ET	0.630	−0.287	−0.593	0.677	−0.332	−0.554
	(0.397)	(0.278)	(0.594)	(0.396)	(0.289)	(0.582)
CER	1.012*	−0.118	−0.191	0.765	−0.286	−0.296
	(0.394)	(0.301)	(0.684)	(0.472)	(0.340)	(0.689)
ET X CER	−2.188**	−0.077	−0.154	−1.806*	0.152	−0.096
	(0.712)	(0.386)	(0.901)	(0.751)	(0.434)	(0.890)
FEMALE				0.018	0.275	−0.631
				(0.414)	(0.224)	(0.441)
POL_PREFERENCE				−0.310	−0.208	−0.108
				(0.192)	(0.109)	(0.214)
ALTRUISTIC-LIKE				1.686***	0.634*	0.524
				(0.482)	(0.252)	(0.507)
Constant	−2.398***	0.887***	3.135***	−2.865***	0.988*	3.535***
	(0.298)	(0.220)	(0.431)	(0.655)	(0.471)	(0.912)
Observations	312	312	312	307	307	307
Pseudo R^2	0.037	0.005	0.016	0.138	0.043	0.035
Chi^2	10.873	3.564	2.808	25.940	24.093	6.224

Table C.3: **GLS random effects regression explaining individually proposed contributions over all eight periods.** The table shows estimates from a GLS random effects panel regression with PROPOSED CONTRIBUTIONS as the dependent variable which represents subjects' proposed contributions in the UC-PGG. Model 1 uses data from the four treatments with unconditional voting while models 2 and 3 analyze data from the two treatments with unconditional voting. ET and CER are dummies for the treatment manipulations. ET takes on a value of 1 if subjects were assigned to groups by conducting the slider task or 0 if they were randomly assigned. CER is 1 if the vote regarding the group change is conducted after the group assignment and 0 if it takes place before the group assignment. FEMALE is a dummy variable and takes on values of 0 for male subjects and 1 for female subjects. POL_PREFERENCE is an ordinal variable from 0 to 6 where 0 means a subject self-identified as rather left–wing, while 6 means a subject self-identified as rather right–wing. ALTRUISTIC-LIKE is a dummy variable taking on values of 0 if subjects' distributional preferences are categorized either as spiteful, kick-down, equality averse, envious or selfish and 1 if their distributional preferences are either kiss-up, inequality averse, maximin or altruistic, respectively. PERIOD is a variable from 1 to 8 and denotes the respective period in the UC-PGG. GROUP is a dummy variable where 1 denotes group HIGH and 0 represents group LOW. MPCR indicates the currently prevailing marginal per capita return of the respective period. CHANGE is a dummy variable for change cohorts denoting 1 and no-change cohorts denoting 0. Clustered standard errors on cohort level (i.e., each cohorts consists of eight subjects and group A and B) are in parentheses. *, **, and *** represent the 5%, 1%, and 0.1% significance level, respectively.

	PROPOSED CONTRIBUTIONS	PROPOSED CONTRIBUTIONS	PROPOSED CONTRIBUTIONS
	(1)	(2)	(3)
ET	−2.395	−5.881	−5.787
	(5.834)	(5.973)	(5.908)
CER	−5.759		
	(6.459)		
ET × CER	7.228		
	(3.794)		
FEMALE	−7.458*	−14.311***	−14.536***
	(3.509)	(4.064)	(4.294)
ET × FEMALE	−6.377	8.233	9.055
	(3.947)	(5.501)	(6.102)
CER × FEMALE	2.702		
	(3.979)		
POL_PREFERENCE	−3.855*	−1.121	−0.935
	(1.633)	(2.346)	(2.285)
ET × POL_PREFERENCE	0.654	1.636	1.326
	(1.810)	(2.708)	(2.611)
CER × POL_PREFERENCE	−1.713		
	(1.855)		
ALTRUISTIC-LIKE	−3.486	4.460	4.160
	(3.218)	(4.824)	(4.736)
ET × ALTRUISTIC-LIKE	−2.653	−5.850	−5.253
	(4.094)	(6.360)	(6.395)
CER × ALTRUISTIC-LIKE	3.729		
	(4.072)		
PERIOD	1.402***	1.330***	1.360***
	(0.203)	(0.328)	(0.354)
GROUP	2.843		−1.976
	(3.017)		(4.533)
MPCR	11.219		−7.702
	(6.747)		(11.831)
CHANGE	1.333		2.822
	(2.158)		(3.728)
Constant	88.301***	85.140***	87.272***
	(6.969)	(4.468)	(8.441)
Observations	3008	1464	1464
R^2 : *overall*	0.072	0.054	0.054
R^2 : *within*	0.042	0.044	0.045
R^2 : *between*	0.088	0.058	0.058

Table C.4: **Ordinary least squares regression explaining subjects' score in the slider task.** The table shows estimates from ordinary least squares (OLS) regression with ET_SCORE as the dependent variable, which represents the amount of correctly positioned slider. Model 1 uses data from the two treatments with unconditional voting while model 2 adds data from one treatment with unconditional voting. The independent variable CER is a dummy for the treatment manipulation. CER is 1 if the vote regarding the group change is conducted after the group assignment and 0 if it takes place before the group assignment. FEMALE is a dummy variable and takes on values of 0 for male subjects and 1 for female subjects. POL_PREFERENCE is an ordinal variable from 0 to 6 where 0 means a subject self-identified as rather left–wing, while 6 means a subject self-identified as rather right–wing. ALTRUISTIC-LIKE is a dummy variable taking on values of 0 if subjects' distributional preferences are categorized either as spiteful, kick-down, equality averse, envious or selfish and 1 if their distributional preferences are either kiss-up, inequality averse, maximin or altruistic, respectively. BELIEF_GROUP_ASSIGNMENT is a variable in percent from 0 to 100 which represents subjects' beliefs about the probability of being in group A. Clustered standard errors on cohort level are in parentheses. *, **, and *** represent the 5%, 1%, and 0.1% significance level, respectively.

	ET_SCORE	ET_SCORE
	(1)	(2)
CER	0.147	0.035
	(0.712)	(0.522)
FEMALE	−3.499***	−2.651***
	(0.812)	(0.685)
POL_PREFERENCE	−0.845	−0.501
	(0.410)	(0.328)
ALTRUISTIC-LIKE	1.610*	1.485*
	(0.770)	(0.595)
BELIEF_GROUP_ASSIGNMENT	0.067**	0.060***
	(0.021)	(0.015)
Constant	14.061***	13.283***
	(2.068)	(1.531)
Observations	186	280
R^2	0.194	0.153
F	7.445	8.470

Table C.5: **Logistic regression explaining subjects' voting behavior in favor or against a group change in treatments with unconditional voting.** The table shows estimates from a logistic regression with VOTE as dependent variable, which is a dummy variable and takes on a value of 0 if subjects vote against a group change and 1 if subjects vote in favor of a group change using unconditional voting described in section 3.3. The three models use different sets of independent variables. Model 1 uses a treatment dummy, model 2 adds subject characteristics, and model 3 adds interaction terms between the treatment dummy and subject characteristics. ET takes on a value of 1 if subjects were assigned to groups by conducting the slider task or 0 if they were randomly assigned. CER is 1 if the vote regarding the group change is conducted after the group assignment and 0 if it takes place before the group assignment. ET X CER is the interaction term for ET and CER and captures the marginal effect. The independent variable FEMALE is a dummy variable and takes on values of 0 for male subjects and 1 for female subjects. POL_PREFERENCE is an ordinal variable from 0 to 6 where 0 means a subject self-identified as rather left–wing, while 6 means a subject self-identified as rather right–wing. ALTRUISTIC-LIKE is a dummy variable taking on values of 0 if subjects' distributional preferences are categorized either as spiteful, kick-down, equality averse, envious or selfish and 1 if their distributional preferences are either kiss-up, inequality averse, maximin or altruistic, respectively. Clustered standard errors on cohort level are in parentheses. *, **, and *** represent the 5%, 1%, and 0.1% significance level, respectively.

	VOTE	VOTE	VOTE
	(1)	(2)	(3)
ET	−0.210	−0.206	−0.717
	(0.426)	(0.443)	(1.007)
FEMALE		−0.391	−0.334
		(0.375)	(0.432)
ET × FEMALE			−0.203
			(0.742)
POL_PREFERENCE		−0.547***	−0.805***
		(0.146)	(0.194)
ET × POL_PREFERENCE			0.439
			(0.281)
ALTRUISTIC-LIKE		0.474	0.949
		(0.481)	(0.798)
ET × ALTRUISTIC-LIKE			−0.730
			(1.001)
Constant	0.547	1.673**	1.991**
	(0.371)	(0.614)	(0.701)
Observations	120	116	116
Pseudo R^2	0.002	0.061	0.074
Chi^2	0.243	17.408	30.437

D Discussion of design features

D.1 Comparing features of the UC-PGG with the VC-PGG

The design of the uniform-contribution public good game (UC-PGG) implemented in this experiment differs in one crucial aspect from the standard voluntary contribution public good game (VC-PGG). In the VC-PGG subjects contribute a voluntary amount of their endowment to the public pool, i.e., there is no mandatory contribution. In contrast, in the UC-PGG subjects propose a contribution and the median of all proposals determines the mandatory, i.e., uniform, contribution of each subject. While specific parameter sets used in the VC-PGG incentivize free-riding on other subjects' contributions, in the UC-PGG everybody is best off if the contribution is maximized. Therefore, the applied mechanism helps to coordinate subjects' preferences toward contributions by effectively removing the free-riding incentives inherent in the classical VC-PGG. The decision for implementing a UC-PGG in this experiment is based on several considerations. First, the uniform-contribution mechanism more closely resembles reality in which citizens as well as migrants have to pay taxes to finance public goods provided by countries. Second, the behavior typically observed in VC-PGG is characterized by falling contributions over time due to strategic considerations, which might severely impact the comparability of choices made in different periods of this experiment. Third, it does not interfere with the purpose of this study, i.e., to investigate subjects' preferences on potential migration. Apart from the varying contribution mechanisms, the UC-PGG as implemented in this experiment and the VC-PGG, share the other essential design elements of public good experiments. Aggregate contributions are multiplied by a factor chosen by the experimenter that usually exceeds 1 to model the additional benefit generated by the public good provision. Further, the increased amount is then distributed equally among all group members. See DeAngelo et al. (2020) for a study that approaches a research agenda related to the one presented here but relying on a standard VC-PGG.

With the design choice for the mechanism that determines contributions in our UC-PGG, we relate to the literature discussing (forced) threshold public good games (Dawes et al., 1986; Van de Kragt et al., 1986; Cartwright and Stepanova, 2017) and minimum-contribution public good games (Andreoni, 1993; Orzen, 2008; Kesternich et al., 2014; Kocher et al., 2016; Keser et al., 2017; Martinsson and Persson, 2019). Even more closely related are the contributions of Gallier et al. (2016) and Kesternich et al. (2018). Gallier et al. (2016) let subjects vote on how to share the burden in a public good game. They test different exogenously and endogenously implemented rules and report that most groups agree on a common scheme in the latter, which increases efficiency. If no agreement is reached, contributions, however, are below those in an exogenously imposed voluntary contribution scheme. Similarly, Kesternich et al. (2018) compare contributions in a VC-PGG to rule-based contributions and observe stable contributions in the latter, which, however, fall short of reaching the maximum efficiency gains. In a series of contributions related to climate negotiations, MacKay et al. (2017) and Schmidt and Ockenfels (2021) suggest that a uniform common commitment (UCC) can increase contributions. In the UCC mechanism proposed by Schmidt and Ockenfels (2021), subjects propose a uniform *minimum* contribution. The lowest of these suggestions becomes the binding contribution of all group members. Conditional on specific conditions, there is a unique Nash equilibrium in

weakly dominant strategies that results in the socially efficient outcome. Comparable to the uniform contribution mechanism (i.e., the median of proposed contributions) in our experiment, this mechanism protects group members against free-riding implying that the selfish behavior coincides with the socially-desirable behavior. Note that these contributions establish a strong link to the existing public goods literature by suggesting and testing approaches to reduce the free-riding incentive inherent in the classical VC-PGG. Because of this link, we decided to label our experiment a uniform-contribution *public good game* although one aspect of the classical version is modified.

D.2 Multiplier, group size, and MPCR

In this section, we discuss the relationship between several important parameters of our experimental design and outline the rationale for choosing the respective numbers. To facilitate this discussion, we summarize important parameter values of the experiment and outline individual subjects' payouts for five levels of median contributions in Table D.6. A similar table is used in the instructions to illustrate the impact of contribution levels on subjects' payout in the experiment. See Appendix A, section A.2 for details. The numbers presented are calculated for all potential combinations of group sizes (2 to 6) and multipliers (1.2, 1.5, and 1.8). Based on these numbers, we, first, discuss the relationship between group size, multipliers, marginal per capita returns (MPCR), and payouts, and, second, our considerations that made us decide for the particular parameter set.

To allow for a clear identification of treatment effects, only one parameter should be changed at a time. Obeying this rule is, however, highly demanding for the experiment presented here. To see this, we start by defining MPCR, which is the ratio between the multiplier and the number of subjects in a group. This number informs about the share a subjects gets from the increased public pool. Keeping the multiplier constant (as we do in this experiment), obviously, the more (fewer) group members, the lower (higher) the MPCR. Thus, a change in the number of group members also changes MPCR implying a violation in ceteris paribus conditions. For our parameter choices, we have MPCR values ranging from 0.20 to 0.75 conditional on group size and multiplier. Still, one can see that, conditional on the contribution level, subjects earn more (less) in absolute numbers if the multiplier is higher (lower). If the multiplier stays constant, subjects' payout are identical, i.e., they are independent of group size. So, for our setting, a higher multiplier will, ceteris paribus, increase subjects' period earnings. Assuming that subjects strive for higher payouts in the experiment, which is also reflected in the incentive scheme, we consider it unlikely that they care much about the MPCR that they get out of the pool.

Instead of changing the multiplier, an alternative approach to modelling the economic impact of migration would have been to implement and communicate it as changes in the MPCR. Implementing this approach, however, would not have solved the violation of the c.p.-condition because keeping MPCR constant while group size changes requires a change in the multiplier (see Isaac and Walker, 1988, for a discussion). Thus, we would have ended up with a larger set of different multipliers that need to be communicated to subjects. Facing this trade-off, we consider changes in the multiplier to be easy to understand for subjects while at the same time

we avoid the need to explain and communicate different MPCR values. We do recognize that there might be a certain influence of MPCR on behavior in VC-PGG when the number of people changes, the uniform-contribution mechanism used in our experiment might mitigate this potential effect. Considering the arguments of this discussion, we decided to change the multiplier accepting changes in the MPCR while at the same time avoiding hard to understand parameter values and changes.

Table D.6: **Overview of important design parameters and payouts conditional on all combinations of group sizes and multipliers.** The table shows for different group sizes (*subj.*) and multiplier realizations (*multi.*) the marginal per capita returns (MPCR), monetary payouts (*payout*), and percentage increases (*%-inc.*) based on endowments (*endow.*) for different levels of median contributions.

						**median contributions**									
						100%		80%		50%		20%		0%	
subj.	group	migr.	multi.	MPCR	endow.	payout	%-inc.	payout	%-inc.	payout	%-inc.	payout	%-inc.	payout	%-inc.
2	LOW	yes	1.5	0.75	10.0	15.0	50%	14.0	40%	12.5	25%	11.0	10%	10.0	0%
3	LOW	no	1.5	0.50	10.0	15.0	50%	14.0	40%	12.5	25%	11.0	10%	10.0	0%
5	HIGH	no	1.5	0.30	20.0	30.0	50%	28.0	40%	25.0	25%	22.0	10%	20.0	0%
6	HIGH	yes	1.5	0.25	20.0	30.0	50%	28.0	40%	25.0	25%	22.0	10%	20.0	0%
6	HIGH	yes	1.2	0.20	20.0	24.0	20%	23.2	16%	22.0	10%	20.8	4%	20.0	0%
6	HIGH	yes	1.8	0.30	20.0	36.0	80%	32.8	64%	28.0	40%	23.2	16%	20.0	0%
